# Quantifying Cooperativity through Binding Free Energies in Molecular Glue Degraders

**DOI:** 10.1021/acs.jctc.5c00064

**Published:** 2025-05-06

**Authors:** Balint Dudas, Christina Athanasiou, Juan Carlos Mobarec, Edina Rosta

**Affiliations:** † Department of Physics and Astronomy, 4919 University College London, London WC1E 6BT, U.K.; ‡ Laboratory of Computational Biology, National Heart, Lung, and Blood Institute, National Institutes of Health, Bethesda, Maryland 20892, United States; § Protein Structure and Biophysics, Discovery Sciences, R&D, AstraZeneca, Cambridge CB2 0AA, U.K.

## Abstract

Molecular glues represent a novel therapeutic modality facilitating the stabilization of protein–protein interactions (PPIs), thus enabling the targeting of previously “undruggable” proteins. We develop a computational procedure to screen for molecular glues using a pathway-independent free energy calculation method for accurately assessing the cooperativity. We employ a combined ligand and protein free energy perturbation (FEP) method to calculate the cooperative effect of a ligand for ternary binding. We study the cooperative binding mechanisms of molecular glue degraders, specifically cereblon (CRBN) modulators targeting Ikaros family zinc finger 2 (IKZF2), a transcription factor implicated in cancer immunotherapy. We present a comprehensive computational protocol for screening large molecular libraries to identify potent molecular glues. By leveraging cooperative binding principles in ternary complex formation, our approach effectively predicts ligand-induced PPIs and their degradation potential. Benchmarking against experimental data for CRBN–Ikaros complexes, our protocol demonstrates high accuracy in identifying superior molecular glues, highlighting L4 and L5 as top performers. Furthermore, our high-throughput screening identified novel candidates from extensive chemical libraries, validated through advanced FEP+ simulations. This study not only underscores the transformative potential of molecular glues in targeted protein degradation but also sets the stage for their broader application across diverse protein targets, paving the way for innovative therapeutic strategies in drug discovery.

## Graphical abstract

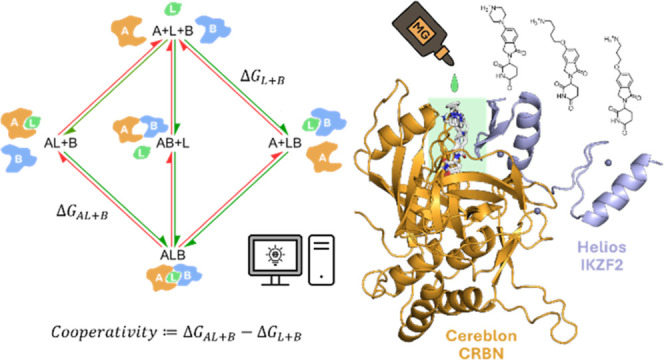

## Introduction

Molecular glues are small molecules that bind at the interface of proteins that otherwise not, or only weakly interact and they stabilize their complex through enhancing protein–protein interactions (PPI). Molecular glues offer a novel therapeutic modality to target currently “undruggable” proteins that lack conventional drug-binding pockets or where access to such sites is hindered. They also offer yet unexploited opportunities in fundamental biochemical research due to their potential to rewire existing cellular pathways through modulating PPIs; either by inducing neo-protein interactions or by pulling proteins out of complexes and guide them to other partners.^1^

In particular, targeted protein degradation became an exciting new paradigm in drug discovery. Small molecular degraders that bind to E3 ligases, such as cereblon (CRBN, a component of the CRL4^CRBN^ E3 ubiquitin ligase) can evoke targeted protein degradation.^2,3^ They make up one of the most prominent scaffolds that consist of thalidomide and its analogues, some of which are already approved drugs (lenalidomide, pomalidomide), in clinical trials (e.g., mezigdomide, galcadomide), or in development pipelines in pharma.^4–6^ In contrast to heterobifunctional molecular degraders (proteolysis targeting chimeras, or PROTACs), molecular glues do not have a linker, consequently they are of lower molecular weight and have molecular properties (increased oral bioavailability, improved cellular permeability) that are more suitable for pharmaceutical dosing.^7^

Structural studies revealed that thalidomide analogues bind to a shallow hydrophobic pocket on the CRBN surface, establishing a scaffold for enhanced PPIs with target proteins (Figure 1). In addition, thalidomide analogs show surprising versatility and selectivity, as demonstrated by the different recruitment of the target proteins IKZF1 and CK1α by two thalidomide analogues, pomalidomide and lenalidomide.^8^ For molecular glue activity, inherent interaction between the protein partners is not a prerequisite as was exemplified for CRBN and its neosubstrates;^9^ instead, molecular glues act by reengineering the protein surface to allow neosubstrate recognition.^10^ Remarkably, target proteins do not need any affinity for the molecular glue itself, as a result, “undruggable”, or even “unligandable” proteins can be targeted for degradation.^3^

**Figure 1:**
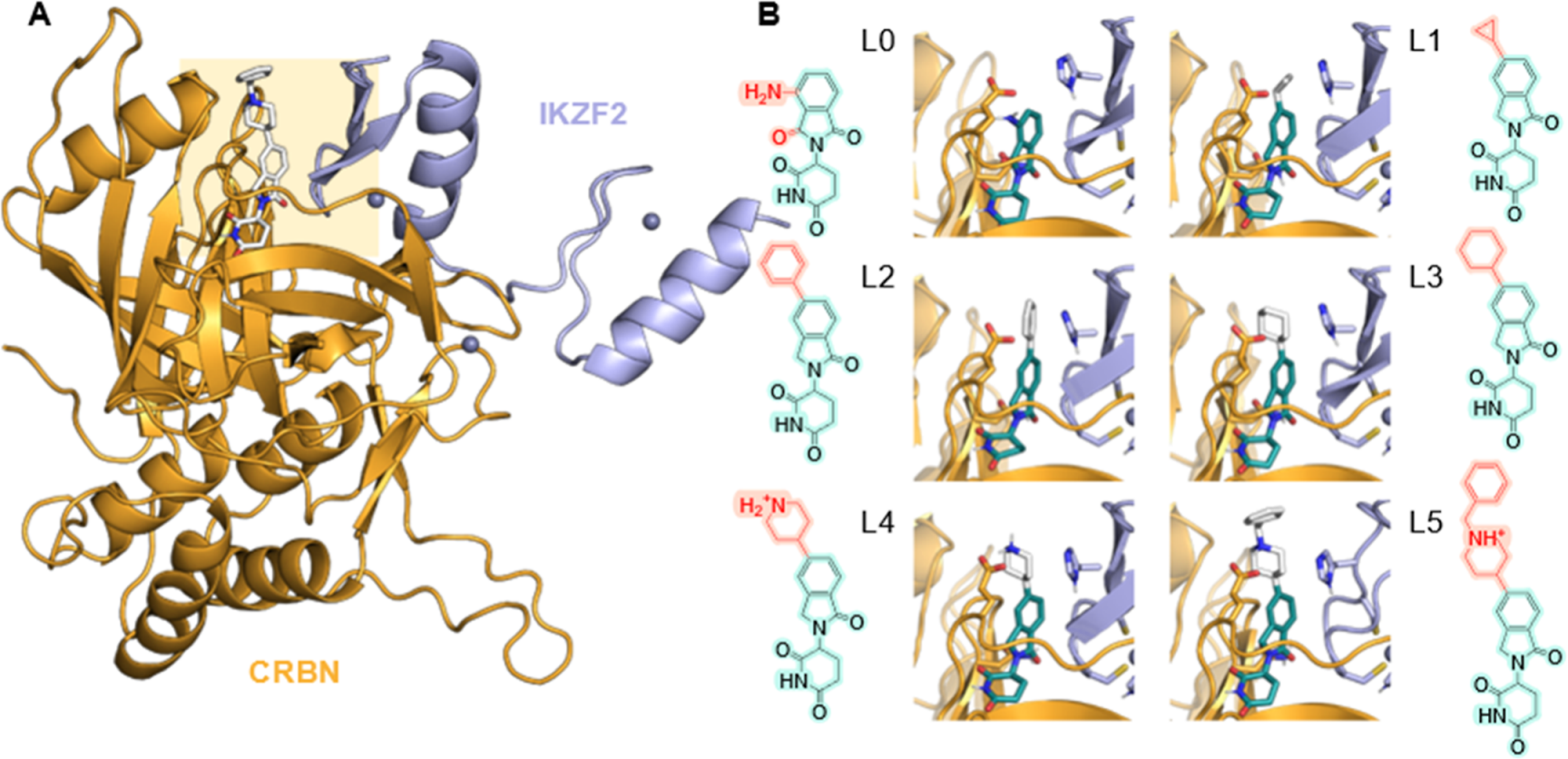
(A) The CRBN/IKZF2:L5 ternary complex of PDB: 8DEY (with zinc fingers 2–3 of IKZF2 in purple ribbons, CRBN in gold and L5 in sticks colored by atom type). (B) Palidomide (L0), and five ligands (L1–L5) from the study of Bonazzi et al.^11^ on the development of a selective IKZF2 degrader (L5) were docked onto the structure of the apo CRBN/IKZF2 complex. Key glutamic acid and histidine residues surrounding the R-group are shown in sticks representation.

Ikaros family zinc finger 2 (IKZF2) is a key transcription factor that is highly expressed in regulatory T cells. IKZF2 is abundantly present in leukemia stem cells^12^ and their loss leads to a reversal of immune-suppressive activity converting Tregs into effector T cells.^13^ Degradation of IKZF2 in Treg cells as well as other T cells could potentially enhance the antitumor immune response, making IKZF2 a promising candidate for cancer immunotherapy. However, targeting zinc finger transcription factors like IKZF2 is challenging due to their largely unstructured nature and the absence of easily targetable binding sites.^14^ IKZF2 has four N-terminal zinc finger domains that are key for DNA binding and stabilization of DNA–protein interactions and further two C-terminal zinc fingers that facilitate homo- and heterodimerization between Ikaros family members.^15^

Different experimental and computational protocols have been established to identify CRBN modulators against various protein targets, most frequently relying on structure–activity relationship models of a limited number of already identified CRBN modulator analogues.^16–20^ Despite the transformative potential of molecular glues in drug discovery and the impressive progress made in recent years, evaluation of large libraries containing several hundreds of thousands of ligands and the denovo development of molecular glues remain a significant challenge.^21^

Cooperative binding has been proposed as a key factor in the activity of molecular glues stabilizing PPIs,^22^ whereby two molecules may form a complex providing a high-affinity binding site for a third molecule. Nevertheless, computational methods are not designed to specifically assess cooperativity. Here, after overviewing and quantitatively describing cooperativity in ternary complex formation, we develop a free energy perturbation-based (FEP) method to calculate cooperativity in a pathway independent manner, incorporating both protein and ligand FEP. We subsequently make use of an efficient computational approximation of cooperativity to establish a protocol for the identification of potent molecular glues in large molecular libraries. To benchmark our protocol, first we test the computational predictions on a set of experimentally tested CRBN modulators targeting IKZF2, after which a large-scale screening is performed whereby novel molecular glues are proposed and are further validated by accurate FEP+ calculations.

## Theory

### Cooperative Binding as the Main Working Hypothesis for Molecular Glue Design

Cooperative binding has been proposed as a key factor in the activity of molecular glues, stabilizing the ternary complex through the multiple interfaces formed between the ligand and two proteins.^22^ A stable ternary complex may be formed, even if the binary interactions between any two isolated components are very weak.^23^ A kinetic mathematical model for the quantification of the cooperativity effect was introduced by Douglass et al.,^24^ describing three-body binding equilibria. Their kinetic definition of cooperativity (α) relates to dissociation rates between two-versus three-component complex formations. Building on this formalism, we use here a pathway independent definition based on thermodynamic relationships.

Three possible paths may exist upon the formation of any ternary complexes (Figure 2), first establishing a dual complex followed by the association of the third component. In the three paths, the following dissociation constants (*K*) are defined for the dual complex formations based on the corresponding on- and off-rates

1 $$K_{d}^{A,L}=\frac{{v_{1}}^{-}}{{v_{1}}^{+}}=\frac{\left[A\right]\left[L\right]\left[B\right]}{\left[AL\right]\left[B\right]}=\frac{\left[A\right]\left[L\right]}{\left[AL\right]}$$

2 $$K_{d}^{L,B}=\frac{{u_{1}}^{-}}{{u_{1}}^{+}}=\frac{\left[A\right]\left[L\right]\left[B\right]}{\left[LB\right]\left[A\right]}=\frac{\left[L\right]\left[B\right]}{\left[LB\right]}$$

3 $$K_{d}^{A,B}=\frac{{r_{1}}^{-}}{{r_{1}}^{+}}=\frac{\left[A\right]\left[L\right]\left[B\right]}{\left[AB\right]\left[L\right]}=\frac{\left[A\right]\left[B\right]}{\left[AB\right]}$$

and for the second steps

4 $$K_{d}^{AL,B}=\frac{{v_{2}}^{-}}{{v_{2}}^{+}}=\frac{\left[AL\right]\left[B\right]}{\left[ALB\right]}$$

5 $$K_{d}^{LB,A}=\frac{{u_{2}}^{-}}{{u_{2}}^{+}}=\frac{\left[LB\right]\left[A\right]}{\left[ALB\right]}$$

6 $$K_{d}^{AB,L}=\frac{{r_{2}}^{-}}{{r_{2}}^{+}}=\frac{\left[AB\right]\left[L\right]}{\left[ALB\right]}$$

**Figure 2:**
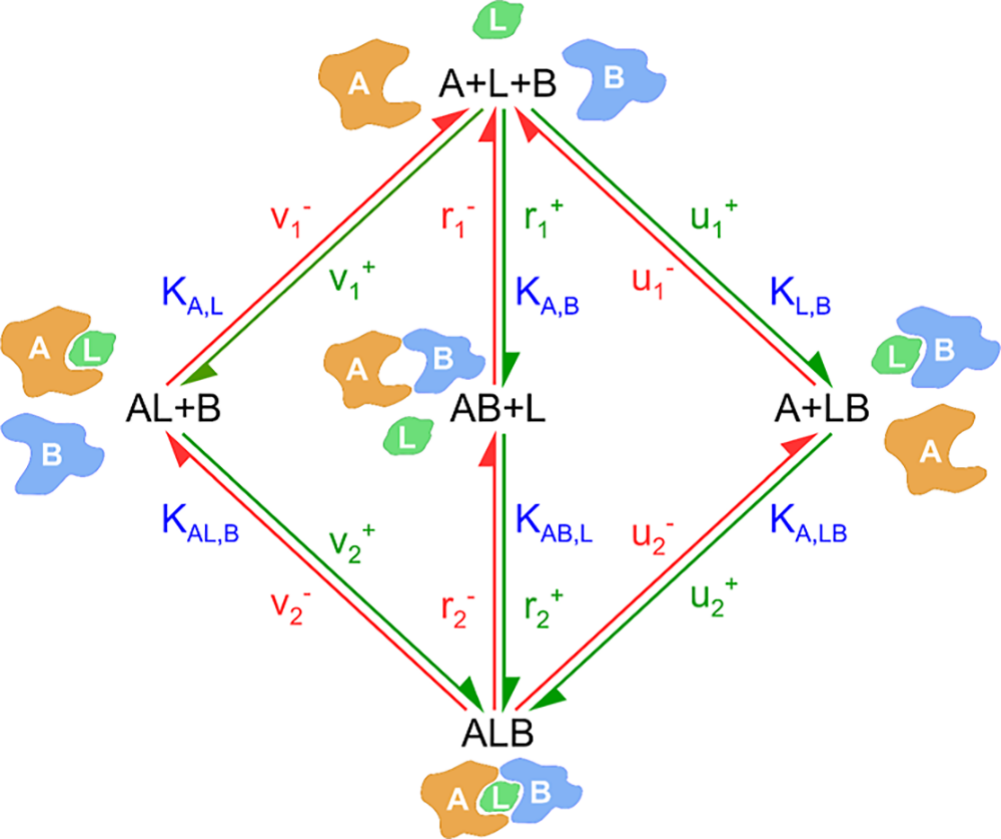
Possible paths leading to the formation of the ternary complex starting from the isolated three components, the two proteins (*A* and *B*) and the ligand (*L*).

From a thermodynamic point of view, cooperativity can be defined as the ratio of the dissociation constants ( $\frac{K_{d}^{AL,B}}{K_{d}^{L,B}}$ or $\frac{K_{d}^{A,LB}}{K_{d}^{A,L}}$ ) for the binding of two components in the presence and absence of the third component

7 $$\alpha_{1}=\frac{K_{d}^{AL,B}}{K_{d}^{L,B}}=\frac{{v_{2}}^{-}{u_{1}}^{+}}{{v_{2}}^{+}{u_{1}}^{-}}=\frac{{u_{2}}^{-}{v_{1}}^{+}}{{u_{2}}^{+}{v_{1}}^{-}}=\frac{K_{d}^{A,LB}}{K_{d}^{A,L}}=K_{d}^{A,L}K_{d}^{L,B}\frac{\left[ALB\right]}{\left[A\right]\left[L\right]\left[B\right]}$$

Pathway specific cooperativity terms, α_2_, α_3_, can also be defined

8 $$\alpha_{2}=K_{d}^{A,L}K_{d}^{A,B}\frac{\left[ALB\right]}{\left[A\right]\left[L\right]\left[B\right]}$$

9 $$\alpha_{3}=K_{d}^{L,B}K_{d}^{A,B}\frac{\left[ALB\right]}{\left[A\right]\left[L\right]\left[B\right]}$$

Indeed, several studies introduced and used different cooperativity terms. Cao et al. used the protein–protein complex as reference (α_2_), the *K*_d_ corresponding to protein complex formation in the absence of a ligand,^22^ whereas e.g. Gadd et al. used α_1_.^25^ Depending on the system of interest, a given path may be considerably more dominant in ternary complex formation than the others, or transitioning along a given path may be energetically very unfavorable and almost entirely nonexistent.

While CRBN/IKZF complexes can form to some extent in the absence of small CRBN modulators, their affinity is very low. Moreover, IKZF itself has little to almost no affinity for existing small-molecule degraders. Consequently, in CRBN ternary complexes, CRBN first binds the ligand, forming a stable CRBN/LIG dual complex that subsequently recruits IKZF or other neo-substrates.^11^ While this is the predominant pathway for CRBN, it may not necessarily apply to all protein–ligand–protein ternary complexes. For instance, when designing molecular glues to enhance existing PPIs, the formation of a dual protein–protein complex may occur at a significantly higher rate, making this alternative pathway more relevant. The choice of cooperativity terms (α_1_, α_2_, or α_3_) to describe the activity of molecular glues depends on which path(s) dominate in the ternary complex formation.

Here, we reformulate cooperativity in terms of binding free energies, which has the advantage of being independent of the paths the system can take. At equilibrium, the molar Gibbs free energy change relates to *K*_d_ as

10 $${\Delta G}^{0}=-RT\;\ln K_{a}=RT\;\ln K_{d}$$

the free energy differences Δ*G*_*A*,*L*_^0^, Δ*G*_*L*,*B*_^0^, Δ*G*_*A*,*B*_^0^, Δ*G*_*AL*,*B*_^0^, Δ*G*_*LB*,*A*_^0^, and Δ*G*_*AB*,*L*_^0^ correspond to the dissociation constants *K*_d_^*A*,*L*^, *K*_d_^*L*,*B*^, *K*_d_^*A*,*B*^, *K*_d_^*AL*,*B*^, *K*_d_^*LB*,*A*^, and *K*_d_^*AB*,*L*^, respectively. Analogously, from a thermodynamic perspective the cooperativity can be expressed as the free energy difference between the binding of two components in the presence and absence of the third component

11 $${\Delta G}_{coop,1}^{0}={\Delta G}_{AL,B}^{0}-{\Delta G}_{L,B}^{0}={\Delta G}_{ALB}^{0}-{\Delta G}_{A,L}^{0}-{\Delta G}_{L,B}^{0}$$

while pathway specific definitions using free energies can be given as

12 $${\Delta G}_{coop,2}^{0}={\Delta G}_{ALB}^{0}-{\Delta G}_{A,L}^{0}-{\Delta G}_{A,B}^{0}$$

13 $${\Delta G}_{coop,3}^{0}={\Delta G}_{ALB}^{0}-{\Delta G}_{L,B}^{0}-{\Delta G}_{A,B}^{0}$$

where Δ*G*_*ALB*_^0^ denotes the free energy difference of the complete ternary complex formation starting from the three isolated components, and Δ*G*_*A*,*L*_^0^, Δ*G*_*L*,*B*_^0^, and Δ*G*_*A*,*B*_^0^ correspond to the dual complex formations. A positive cooperativity is present if Δ*G*_coop_^0^ < 0 (equivalently α > 1), no cooperativity exists if Δ*G*_coop_^0^ = 0 (α = 1), and negative cooperativity (or inhibition, such as for protein–protein interaction inhibitors) exists if Δ*G*_coop_^0^ > 0 (α < 1).

When comparing the cooperative effects of different ligands, relative cooperativity can be defined as

14 $${\Delta \Delta G}_{coop,1}^{0}={\Delta \Delta G}_{ALB}^{0}-{\Delta \Delta G}_{A,L}^{0}-{\Delta \Delta G}_{L,B}^{0}$$

which in terms can be calculated e.g. from relative ligand binding FEP simulations.

We define a computationally efficient approximate cooperativity estimate for the screening of large ligand libraries as the differences in pairwise interaction energies between the components in the ternary vs the dual complexes

15 $${\Delta G}_{coop}\approx {\Delta IntE}_{AL}\left(AL\to ALB\right)+{\Delta IntE}_{LB}\left(LB\to ALB\right)+{\Delta IntE}_{AB}\left(AB\to ALB\right)$$

where IntE_*AL*_, IntE_*LB*_, and IntE_*AB*_ are the interaction energies between *A* and *L*, *L* and *B*, and *A* and *B*, respectively. We found that IntE_*AB*_ requires considerable simulation lengths of multiple runs to converge, we present our results both with and without these contributions for comparison.

## Results and Discussion

To establish a protocol for the design of molecular glues that act as degraders, we first focused on available experimental complex association data between CRBN and IKZF induced by different pomalidomide derivatives. Bonazzi et al. pursued a recruitment-guided medicinal chemistry campaign which lead to the identification of a selective IKZF2 degrader, while testing 6 pomalidomide derivatives against both IKZF1 and IKZF2.^11^ We employed different computational tools to evaluate the stability of ternary complexes and compared their results against the available experimental data on IKZF/CRBN cellular recruitment assays. Our analysis relied on the energetics originating from nonbonded energy contributions (Interaction Energy, IntE) during molecular dynamics simulations, binding enthalpy using molecular mechanics, general Born surface area (MMGBSA), and binding free energy calculated using ligand perturbation simulations (FEP+). For the detailed protocols see the Methods section. To assess the stability of our simulations, readers are encouraged to refer to the monitored rmsds in Figures S2–S4.

### Binding Affinities in the Ternary Complex

For a small molecule to act as a good degrader it is an important prerequisite that it can induce the formation of a stable ternary complex, even though the complex formation alone does not necessarily translate to degradation. As an example, experimentally it was observed that L1, L4–L5 against IKZF1 and L2–L3 against IKZF2 all have a maximum recruitment activity above 500% with respect to the glue-free complex recruitment, yet IKZF degradation could not be observed for them even in the regime of very high ligand concentrations (>50 μM).^11^ However, those glues exhibiting an exceptionally high capability of ternary complex formation (recruitment activity >1000%) were able to degrade the Ikaros factors (L0 against IKZF1 and L4–L5 against IKZF2). In our analysis, we focused on the complex formation induced by molecular glues and aimed to distinguish those molecules with outstanding capacity to stabilize the ternary complexes.

The experimental recruitment results show that L1 is the least active against IKZF2 compared to all other ligands, and L5 has the greatest activity (Figure 3A). L2 has a similar ability to stabilize the complex as L0, whereas L4 has a maximum activity not much inferior to L5, yet only in the presence of much higher ligand concentrations. All methods managed to rank L4 and L5 in front of the rest (Figure 3B–D). The IntE and the MMGBSA analysis predicted L4 to be superior to L5, whereas the FEP+ analysis ranked L5 as the best glue in agreement with the experiments. Interestingly, in all three cases the performance of the reference L0 seems to be slightly underestimated, and all L1–L5 are predicted to be better glues than L0, whereas experimentally L1 was observed to perform worse than L0. Among L1–L3, the three methods do not establish a clear consensus ranking.

**Figure 3:**
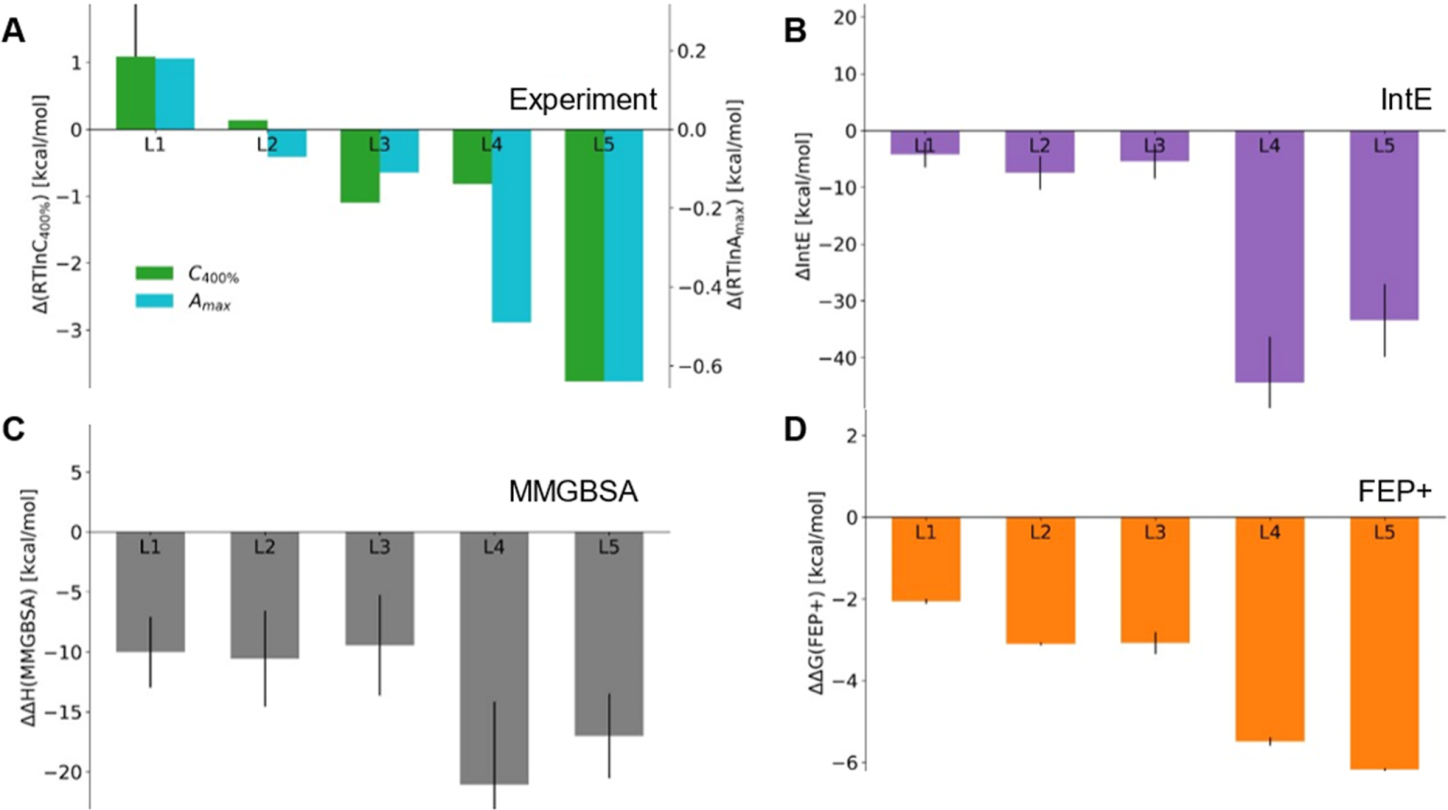
Relative binding affinity to CRBN/IKZF2 of L1–L5 with respect to L0. (A) Experimental *A*_max_ values (cyan) and concentrations corresponding to 400% IKZF2 CRBN recruitment (green), transformed to free energy-like quantities (by taking *RT* ln *C*). The experimental data originates from the recruitment-guided medicinal chemistry campaign by Bonazzi et al.^11^ (B) Interaction energy and (C) MM-GBSA enthalpy calculated from the 100 ns-long MD simulations of the ternary complexes. (D) Binding free energy calculated by FEP+ ligand perturbation simulations.

Even though all ligands L0–L5 are capable of inducing IKZF2 recruitment—ranging from 340% to 1350% maximum recruitment activity with respect to the ligand-free complex formation—and the task here is to distinguish between ligands of comparable performances, all three computational analyses managed to clearly differentiate between the best-performing ligands (L4 and L5) and the rest, solely based on the energetics observed in the ternary complexes.

We evaluated cooperativity in our simulations for L0–L5 and compared the results with experimental complex association data. In agreement with the binding affinities discussed previously, all computational results agree that L1 is the least potent ligand among L1–L5 (Figure 4), and all methods ranked L4 and L5 in front of the rest based on their cooperativity. This agreement is not straightforward, as favorable binding affinity in a ternary complex does not necessarily imply a free energy gain nor does it explain its extent upon the formation of the ternary complex compared to the dual complexes. Even for L1, the FEP+ simulations predict it to have stronger cooperativity than the reference, L0 (Figure 4C), which may be a result of underestimating L0 as discussed previously. Between L4 and L5, the IntE and the MMGBSA analysis predicted L4 to exhibit a stronger CE as opposed to the FEP+ analysis that predicted L5 having the strongest cooperativity, in agreement with the experimental recruitment data. The results shown in Figure 4A,B simplify the cooperativity estimation by not including the ΔIntE_*AB*_ term in the ternary complexes. A good estimate of IntE_*AB*_ requires multiple runs, and the values have a considerable standard deviation (Figure S1). We found that no significant difference exists between the IntE_*AB*_ in the dual protein-complex and in the ternary complexes with the exception of L4 where a strong salt bridge is missing between E137_IKZF2_ and R373_CRBN_ that weakens the interactions between the two proteins. Even though all six ligands are capable of enhancing IKZF2 recruitment to some extent, the CE analysis is capable of differentiating between the best-performing L4 and L5, and the least favorable L0 and L1.

**Figure 4:**
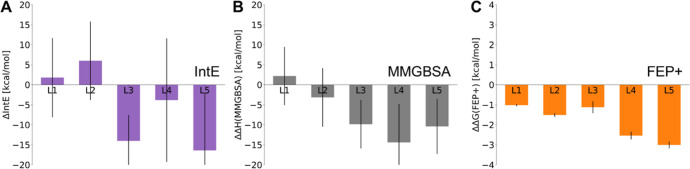
Relative cooperativity effects in the CRBN/IKZF2/LIG complexes with pomalidomide as reference. Cooperativity effects calculated by (A) interaction energy, (B) MM-GBSA, and (C) FEP+.

### Molecular Basis of Differences between IKZF Degraders

To better understand the origin of the differences in gluing performances or “glueability” observed between the different ligands, we analyzed the interactions in the ternary complexes on the residue level. IntE averaged over the production MD simulations were calculated between L0–L5 and the CRBN/IKZF2 residues (Figure 5). We found that largest difference in CRBN binding originates from the presence of a positive charge in L4 and L5 that brings in close contact the negatively charged E377_CRBN_, a forceful interaction which is absent for L0–L3. The observed >18.5 kcal/mol difference between the charged and uncharged ligands interacting with E377_CRBN_ explains how the charged ligands are much more firmly glued to CRBN. We note, however that electrostatic interactions can be overestimated,^26^ nevertheless this remains a key factor even with using a distance dielectric constant of 2 decaying with *R* ^–2^. Other differences are minor compared to E377_CRBN_, e.g. with H353_CRBN_ (strongest IntE with L5: −4.4 ± 0.6 kcal/mol while weakest with L0: −1.7 ± 0.6 kcal/mol). All ligands establish strong interactions with W380_CRBN_ (−8.9 ± 0.8 to −9.3 ± 0.7 kcal/mol) and W386_CRBN_ (−6.5 ± 0.8 to −7.4 ± 0.9 kcal/mol), as well as somewhat weaker interactions with N351_CRBN_ (−4.2 ± 0.8 to −6.8 ± 1 kcal/mol), H378_CRBN_ (−4.4 ± 1.1 to −6.0 ± 0.8 kcal/mol), and P352_CRBN_ (−4.3 ± 0.6 to −4.8 ± 0.7 kcal/mol).

**Figure 5:**
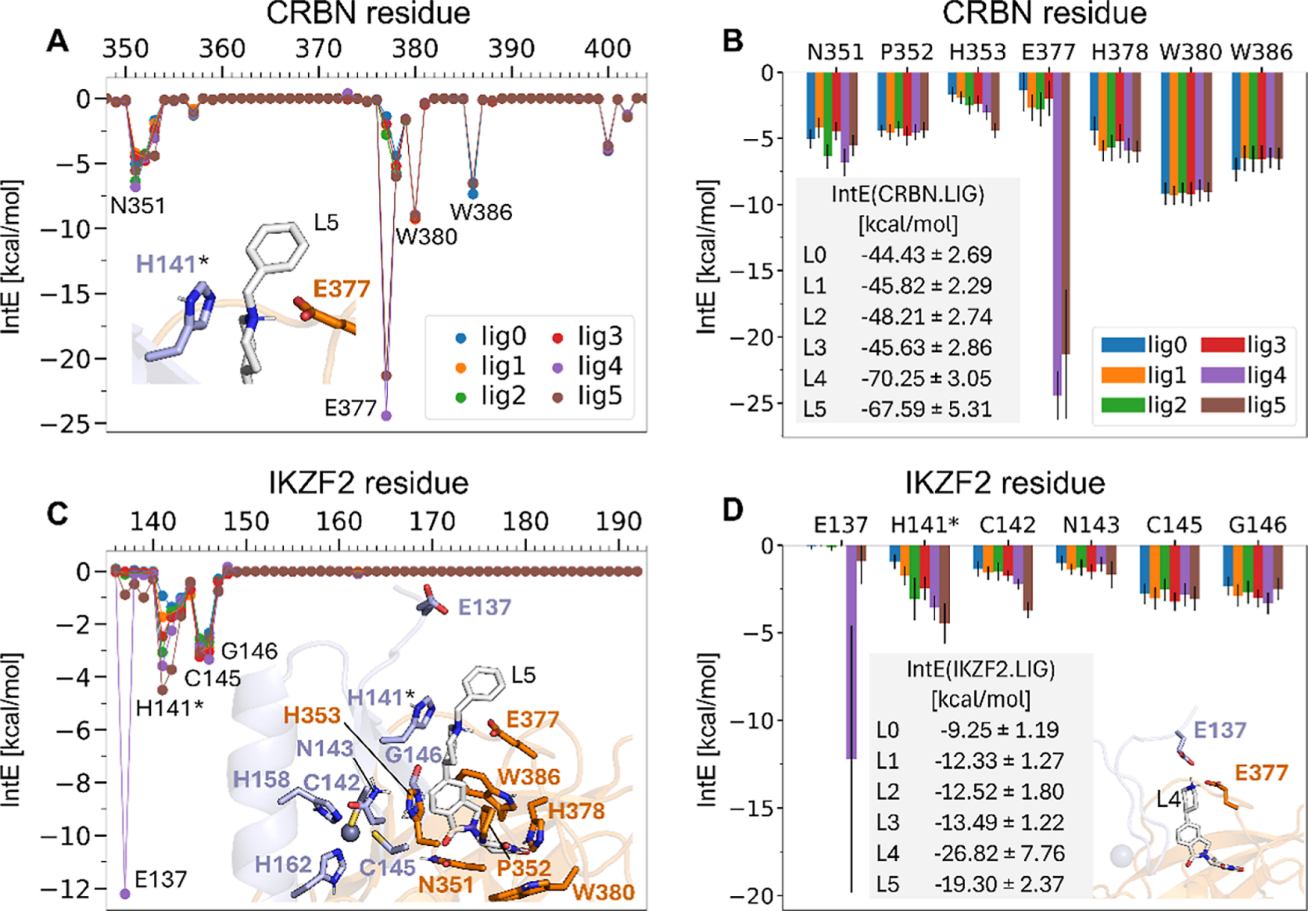
Glueability analysis of interactions between the ligands and CRBN (A,B) or IKZF2 (C,D). Interactions are calculated on the residue level, and are averaged over the MD simulation conformations sampled. The total IntEs between CRBN and the ligands are summarized in the table in panel B, and between IKZF2 and the ligand in panel D. In the structural figures CRBN and the corresponding residue notations are in orange, IKZF2 in light blue. H141* is highlighted as it is the main substitution between IKZF1 and IKZF2 from Q to H.

For an efficient molecular glue, strong interactions with the other binding partner are also essential, the IntE between L0–L5 and the IKZF2 residues are shown in Figure 5C,D. L4 differed significantly in the IntE values with E137_IKZF2_ as compared with the other ligands. This interaction is 11.3 kcal/mol more favorable compared with the also charged L5, and is negligible for the uncharged L0–L3. This interaction is established at the loose N-terminal end of the IKZF2 which gets in close contact with the charged amine group of L4, whereas the additional bulky benzyl group in L5 obstructs the formation of this strong salt bridge. However, as discussed previously, E137_IKZF2_ forms a salt bridge with R373_CRBN_ for the other ligands, which is missing for L4. We hypothesize furthermore that such a rearrangement of IKZF2 may not be possible in the full-length protein and therefore this interaction might lead to the overestimation of the molecular glue performance of L4 in our simulations.

Interestingly, interactions with H141_IKZF2_ differentiate well between the ligands, ranging from −0.9 kcal/mol for L0 to −4.5 kcal/mol for L5, and the ranking of the ligands also agrees well with their overall glue performance observed in the experiments, ranking L5 on top followed by L4, while L0 and L1 the worst. H141_IKZF2_ is the residue that is replaced by glutamine in IKZF1, and is crucial for designing selective IKZF2 degraders. C142_IKZF2_ also favors interactions with L5, followed by L4 with 1.5 kcal/mol less favorable IntE. Interactions with N143_IKZF2_, C145_IKZF2_, and G146_IKZF2_ are also present, those for all ligands.

### Selectivity for the IKZF2 Isoform

For degraders of the Ikaros family, it may be beneficial to develop selective degraders only targeting a specific member. It has been shown that L0 (pomalidomide) is active against IKZF1 and it does not degrade IKZF2, whereas L5 was developed as a selective IKZF2 degrader sparing the degradation of IKZF1.^11^ We performed FEP+ residue perturbation simulations in the presence of L0–L5 to determine the ligand preferences of the CRBN–Ikaros complexes. We found—in agreement with experiments—that L0 binds more favorably to IKZF1, whereas L2–L5 to IKZF2 (Figure 6). FEP+ predicted even L1 to favor IKZF2, in contrast to experiments that showed higher affinity toward the IKZF1 ternary complex, yet without inducing IKZF1 degradation. Interestingly, the FEP+ calculations found L5 as showing the largest binding free energy difference between IKZF2 and IKZF1 complexes emphasizing its selectivity, yet experiments identified L3 with the largest difference.

**Figure 6:**
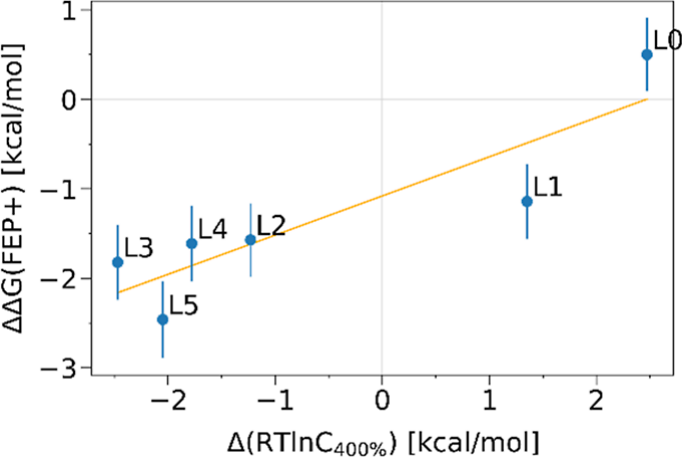
Correlation between ligand binding affinities to IKZF2 and IKZF2­(H141Q) determined by experiments and retrieved by FEP+ simulations. The experimental values are transformed to obtain free energy-analogous quantities (by taking *RT* ln *C*).

We performed simulations on IKZF2­(H141Q) mimicking IKZF1 (there are a total of four residue differences between IKZF2 and IKZF1 at the zinc fingers 2 and 3). However, the comparison of the energetics in the IKZF2­(H141Q) complexes did not capture the selectivity identified with the FEP+ residue perturbation simulations (Figures S5 and S6). All of the IKZF2­(H141Q) simulation results suggested that both L4 and L5 are more potent molecular glues than L0. We speculate that additional differences between IKZF2 and IKZF1 beyond residue 141 may play a crucial role in IKZF1 recruitment.

### Virtual Screening Campaign for the Discovery of IKZF2 Degraders

Next, we aimed to identify new potent molecular glues targeting IKZF2 (Figure 7). We performed a substructure search (see Methods) for ligands that share similarities with L0–L5 to ensure some interactions with CRBN and guide the positioning of new hits.

**Figure 7:**
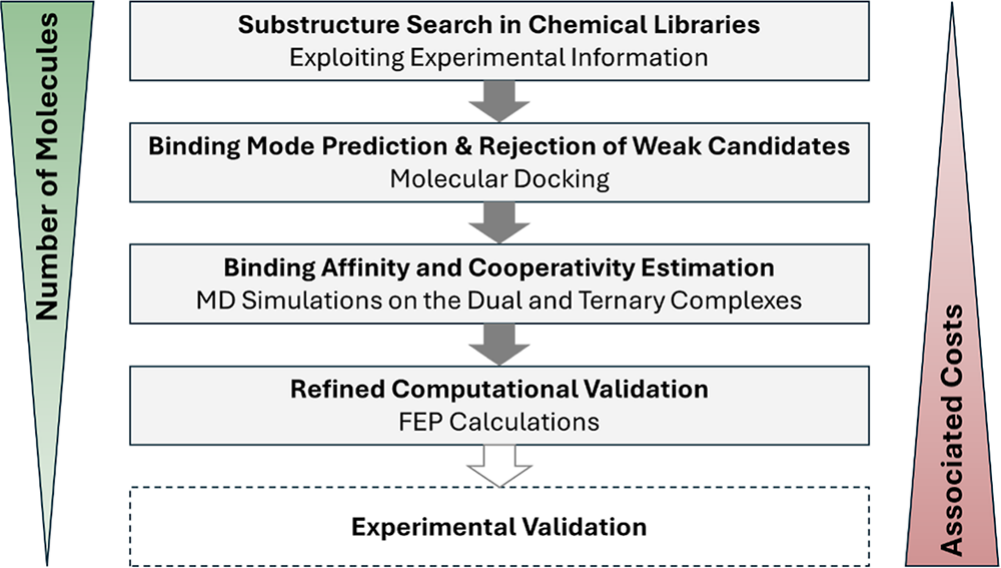
Flowchart of the proposed high-throughput ligand screening pipeline for identifying molecular glues. The process begins with a substructure search in large chemical libraries, guided by experimentally identified molecular glue cores. Retrieved molecules undergo molecular docking to predict their binding modes and eliminate weak candidates. Promising compounds are then assessed for binding affinity and cooperativity by running MD simulations on both protein–ligand dual and ternary complexes. Top candidates undergo refined computational validation using FEP calculations for higher accuracy. Finally, the best hits are proposed for experimental validation.

The retrieved molecules were prepared and docked to the CRBN.IKZF2 complex using Schrödinger Maestro and Glide (see Methods section for details). We then performed MD simulations on the CRBN/LIG, the IKZF2/LIG, and the CRBN/LIG.IKZF2 complexes, and calculated the ligand binding affinities in the ternary complex as well as the CE (Figure 8). In addition to calculating the absolute IntE and CE that correspond to enthalpic contributions, in order to account for entropic losses to some extent, we recalculated the results normalized by the molecular mass of the ligands (Figure S7).

**Figure 8:**
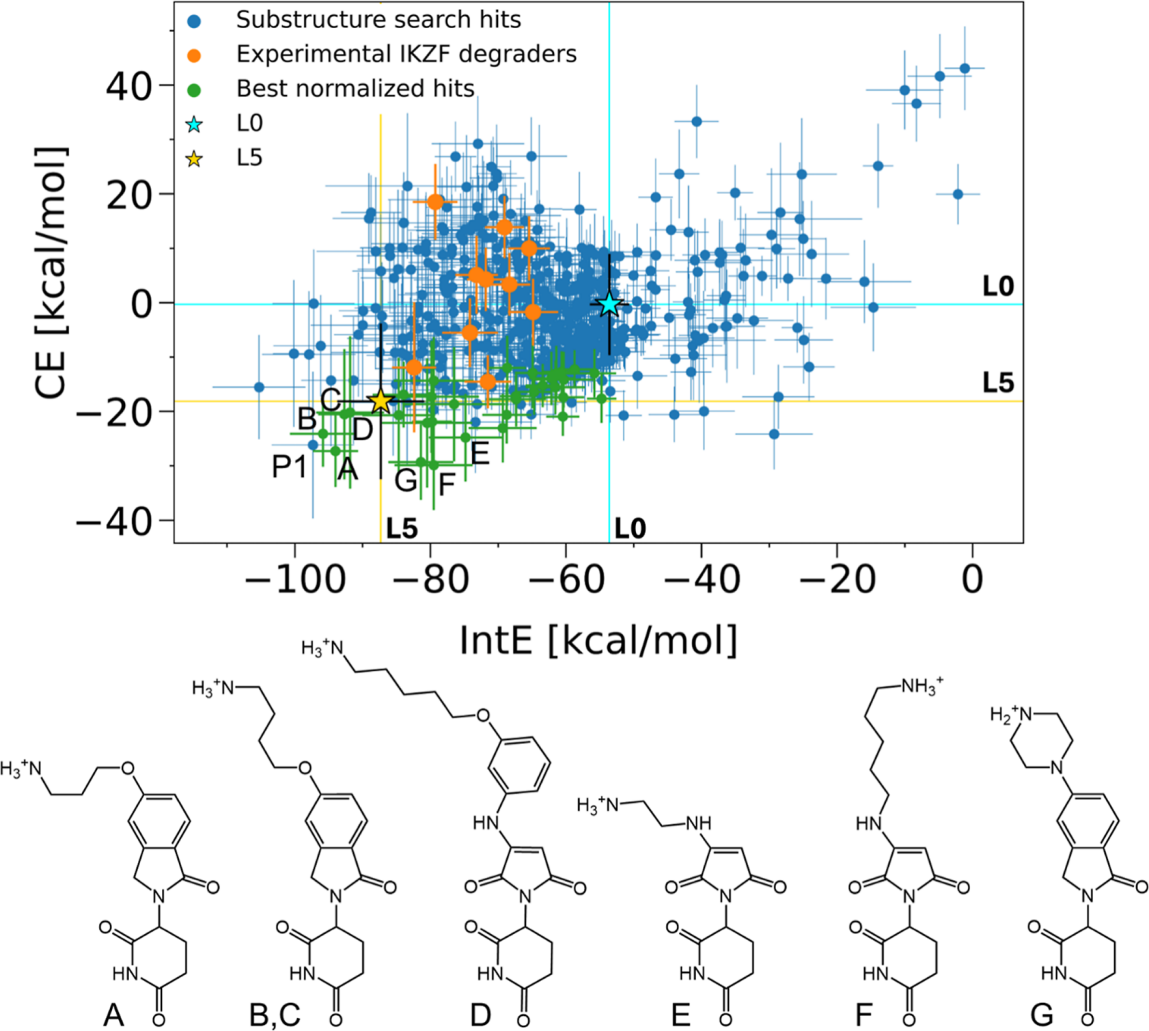
Predicted cooperativity effect (CE) and interaction energy (IntE) for substructure search hits, experimental degraders (orange), and reference ligands L0 and L5 (cyan and gold stars, respectively). Molecules outperforming L5 in IntE and CE after molecular weight normalization are highlighted in green. Experimental degraders targeting IKZF proteins (IKZF1-3) were sourced from the MedChemExpress database (https://www.medchemexpress.com). Molecules A–D are predicted to exhibit both improved IntE and CE compared to L5, with B and C representing the same molecule in different binding modes. Molecules E–G, along with A and B, rank among the top five molecules after molecular weight normalization. One of the top-ranked molecules was omitted from further FEP analysis due to its large size (marked by P1).

We found that 53% (316/596) of the tested compounds have both more favorable unnormalized IntE in the ternary complex and unnormalized CE than L0, while 26.5% (158/596) were identified to be more favorable in the normalized case. As L5 is the most potent molecular glue in our reference data set, we also compared our results to it. If not normalized, 5 data points were found to be superior even to L5, corresponding to 4 distinct molecules (Figure 8, one molecule had two different binding modes included). With normalization by the molecular masses, this increases to 32 (with 30 distinct molecules), including 3 molecules from the unnormalized best hits (Figures 8, S7 and S8). Notably, two of the top five hits in the normalized analysis also rank among the top hits without normalization. Interestingly, while all top unnormalized hits carry a +1 charge similar to L5, 16 out of 32 normalized hits are neutral. One top-ranked molecule from the unnormalized analysis was omitted from further FEP analysis due to its large size (Figure S9, marked by P1 in Figure 8). The size of this ligand is comparable to heterobifunctional PROTACs (e.g., NX-2127 ^27^) but the aim of this study was to identify small molecular glues that possess better pharmacokinetic properties. The best hits retained by this computationally fast protocol were then further analyzed (molecules A-G in Figure 8) to identify crucial contacts within the ternary complexes.

### Interactions of the Best Hits in the Ternary Complexes

To further investigate the molecular basis of the predicted top hits’ gluing capacity, we analyzed their residue-level interactions with CRBN and IKZF2 in their ternary complexes during MD simulations (Figure 9, interactions of further top candidates are shown in Figure S10). Since candidates were identified through a substructure search, they share a common core with L0–L5. As expected, the best candidates retained key interactions with CRBN, including contacts with E377_CRBN_, W380_CRBN_, W386_CRBN_, H378_CRBN_, N351_CRBN_ P352_CRBN_, H353_CRBN_, and W400_CRBN_. Notably, five candidates (molecules P1, A–C, and G) exhibited even stronger interactions with negatively charged E377_CRBN_ than the reference L5, further anchoring them to CRBN. Additionally, four candidates (A–C and G) showed enhanced interactions with N351_CRBN_, while molecules A–C even formed hydrogen bonds with H357_CRBN_—an interaction that is negligible in L0–L5.

**Figure 9:**
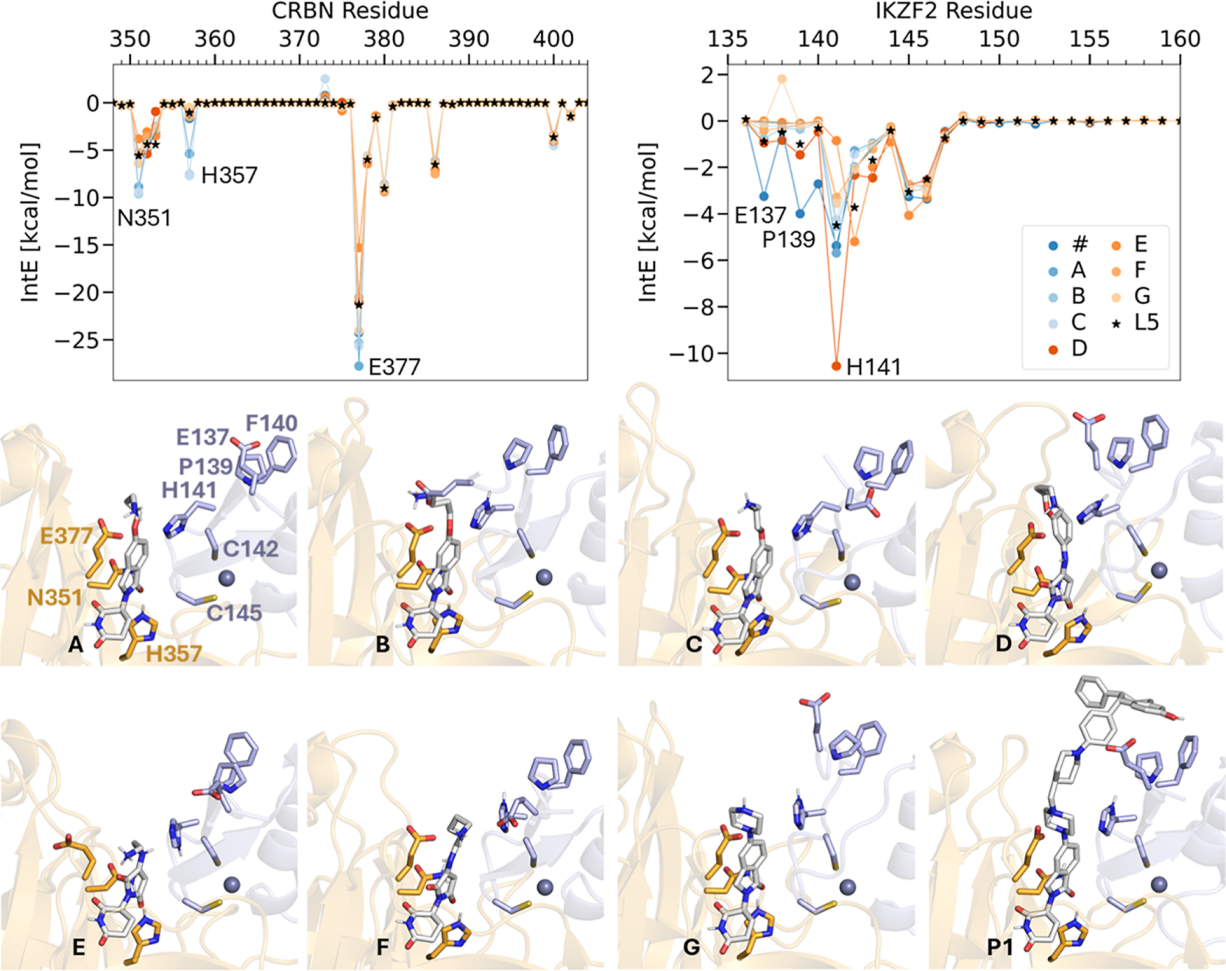
Residue-level interactions between the top glue candidates and CRBN and IKZF2. Interactions with the most significant deviations from the reference L5 are annotated (top). Reported values represent averages from the MD production runs. The binding modes of the best candidates are depicted as observed at the end of the MD simulation (structural panels).

The top glue candidates also preserved key interactions with IKZF2, specifically with H141_IKZF2_, C142_IKZF2_, C145_IKZF2_, and G146_IKZF2_. Notably, three candidates (P1, A, and D) exhibited stronger interactions with H141_IKZF2_ than the reference L5. This residue is replaced by glutamine in IKZF1 and is crucial for designing selective IKZF2 degraders. In particular, molecule D showed a significantly more favorable interaction—over 6 kcal/mol stronger than L5—by orienting its positively charged amine group toward the deprotonated Nε of H141_IKZF2_.

Additionally, the bulky PROTAC-like candidate (P1) demonstrated enhanced interactions with E137_IKZF2_, P139_IKZF2_, and F140_IKZF2_ compared to L5. A strong interaction also existed between the experimental L4 and E137_IKZF2_ (Figure 5), which hindered the formation of a key salt bridge between E137_IKZF2_ and R373_CRBN_. While this salt bridge was absent in the initial experimental complex (PDB: 8DEY), it formed during extended MD simulations for L0–L3, L5, and one glue candidate (molecule F) in its short MD production run. Notably, interactions with P139_IKZF2_ and F140_IKZF2_, which were nearly absent in L0–L5, were significantly improved in the bulky candidate.

### FEP Analysis of the Top Hits

We further analyzed the predicted top hits using FEP simulations, similar to our previous analysis on L0–L5. We included molecules that demonstrated both improved IntE and CE compared to L5, either in the normalized or unnormalized analysis (Figure S8 and Table S1). Relative binding affinities (ΔΔ*G*) and relative cooperativities (ΔCE) were calculated with respect to L0.

From our FEP calculations, we identified 17 candidate molecules with more favorable Δ*G* and CE values than L0. Although L5 remained the top-ranked glue, 7 candidates (among which four molecules: G, B, C, and A from Figure 8) outperformed L0–L3 (Figure S11). Notably, four out of the seven candidates that were best ranked using our computationally fast protocol (shown in Figure 8) were also ranked among the best 5 molecules identified by the more accurate but computationally very expensive FEP simulations. While our analysis confirmed L5 to be a very potent molecular glue (ranking it highest), L5 was originally developed through a recruitment-guided medicinal chemistry campaign and is not present in the screening databases. In fact, the top predicted candidate has a ΔΔ*G* of −4.3 ± 0.6 kcal/mol and ΔCE of −2.2 ± 1.0 kcal/mol with respect to L0, while L5 had a ΔΔ*G* of −6.4 ± 0.7 kcal/mol and ΔCE of −3.3 ± 1.0 kcal/mol. Interestingly, this candidate that ranked highest in our screening campaign (molecule G in Figures 8 and 9 and CSBRL0004 in Table S1) highly resembles L4, with only a nitrogen replacement within their charge-carrying aliphatic ring. As a result, our top hits could also be possible further improved by molecular optimization.

Our virtual screening protocol, especially by extending it to larger chemical libraries, can identify candidates that after further optimization could achieve potency comparable to L5 in targeting IKZF2. Furthermore, the presented protocol not only ranks candidates based on glueing capability, but it also enables a detailed residue-level molecular analysis to guide design to further improve interactions in the ternary complex. Our protocol is readily transferable to other PPIs, and can be particularly useful for PPIs where experimental molecular glues have not yet been identified.

## Conclusions

In this study, we developed and validated a robust computational framework based on free energy calculations for the identification of potent molecular glue degraders targeting IKZF2 from extensive molecular libraries. By focusing on the principle of cooperative binding within ternary complexes, our approach offers a scalable solution to the longstanding challenge of screening vast chemical spaces for effective molecular glues.

Our protocol’s accuracy was rigorously benchmarked against experimental data involving CRBN modulators, demonstrating its capability to reliably predict the stabilization and degradation potential of molecular glues. Notably, our computational analyses distinguished L4 and L5 as the most effective ligands, aligning with experimental observations. Furthermore, the high-throughput screening of large molecular libraries unearthed novel candidate molecules, which were subsequently validated through advanced free energy perturbation (FEP+) simulations.

Despite these advancements, experimental data on ternary complex formation remains sparse in the literature, and the lack of negative data presents a challenge in fully validating computational predictions. However, experimental data on the selectivity between IKZF1 and IKZF2 allowed us to address some aspects of negative data. While experimental validation was beyond the scope of this study, we envision that future large-scale screenings and predictions can further refine our computational scheme. Furthermore, screening campaigns targeting CRBN neo-substrates can be also extended beyond the shared common core of L0–L5, potentially discovering novel scaffolds for future CRBN modulators.

The implications of our findings extend beyond the immediate scope of IKZF2 degradation. Our strategy does not require the estimation of the most dominant pathway for the binding kinetics, rather it is capable to handling these concurrently. This computational strategy could help enable the discovery of molecular glues across a broad spectrum of protein targets and PPIs, offering a versatile tool for drug discovery.

Our study lays a solid foundation for the computational identification of molecular glues, opening new avenues for targeted protein degradation therapies. The potential applications of this approach in denovo design and its scalability for large and diverse libraries underscore its transformative impact on the field of drug discovery.

## Methods

### System Building

Ternary (CRBN/IKZF.LIG) and dual (CRBN/LIG or IKZF/LIG) complexes were built starting from the crystal structure of CRBN–DDB1 (DNA damage-binding protein 1) bound to IKZF2 (zinc fingers 2 and 3) and the molecular glue DKY709 (LIG5), PDB: 8DEY.^11^ As DDB1 is not involved in the binding of molecular glues that stabilize the PPI between CRBN and IKZF, DDB1 was omitted from our simulations. Ligands L0–L4 were positioned in the complex by overlapping their shared substructure on LIG5 after 100 ns equilibration of the CRBN/IKZF/LIG5 complex. Protonation states of the titratable groups were assigned after analysis with PROPKA at pH 7 ± 1 with the Protein Preparation Workflow in Maestro (Schrödinger, Inc.).^28^ The H-bond network was also optimized by reorienting hydroxyl and thiol groups, water molecules, amide groups of asparagine (Asn) and glutamine (Gln), and the imidazole ring in histidine (His) and predicting protonation states of histidine, aspartic acid (Asp) and glutamic acid (Glu) and tautomeric states of histidine. A restrained energy minimization was performed until the rmsd of the heavy atoms relative to the unminimized structure exceeded the 0.3 Å threshold. Glutamates 146^CRBN^ and 311^CRBN^ were protonated. LIG4 and LIG5 had an overall +1 charge, whereas LIG0-3 were neutral.

Correct coordination of the zinc-fingers (4-coordinated zinc metal centers) was ensured by adapting the zinc AMBER force field (ZAFF) parameters,^29^ introducing covalent bond definitions between the zinc center and the coordinating residues. Ligand parameters were determined using antechamber^30^ with the AM1-BCC charge model^31,32^ and the general AMBER force field 2 (GAFF2) atom types.^33^ The complexes were solvated using the TIP3P^34^ water model using CHARMM-GUI,^35^ the NaCl concentration was set to 0.15 M.

For all the simulations the all-atom additive CHARMM C36m force field^36^ was used. Simulations were performed by NAMD.^37^ First, the systems were energy minimized for 10,000 steps using the conjugate gradient method starting from the solvated complexes built using the 100 ns-long equilibrated CRBN/IKZF/LIG5 complex. All systems were equilibrated at 300 K for an additional 2 ns in an *NVT* ensemble. This was followed by 100 ns-long *NPT* production runs at 1 atm pressure. Langevin dynamics was used with an integration time step of 1 fs, a damping coefficient of 1 ps^–1^, a piston oscillation period of 50 fs, and a piston oscillation decay time of 25 fs. For the energy calculations, the dielectric constant was set to 1. The particle mesh Ewald (PME) method was used to calculate the electrostatic interactions with a maximum grid spacing of 1 Å having the order of 6. The cutoff for nonbonded interaction was set to 12 Å and the switch distance to 10 Å.

### Free Energy Perturbation (FEP) Calculations

FEP calculations were performed with the FEP+ tool of the Schrödinger Suite 2023-4.^38^ Before the FEP calculations, the protein complexes and the ligands were prepared with the Protein Preparation Workflow and LigPrep tools, respectively, at pH = 7 ± 1. Then a Glide docking^39,40^ was used with core constraints to LIG5 from the 8DEY crystal structure, to ensure correct placement of the pomalidomide core. The Force Field Builder panel was used to calculate any missing dihedral angles on the ligands. The FEP+ panel was used to create a graph of mutations and define the “hot” regions for the replica exchange with solute tempering (REST2) simulations. Each perturbation edge comprised a solvent and a complex perturbation leg. For each leg, the system was solvated in an orthorhombic SPC water box and neutralized with NaCl. An ionic strength of 150 mM NaCl was used for charged perturbations. The OPLS4 force field was used. Neutral and charged perturbations were run using 12 and 24 lambda windows, respectively. The default FEP+ simulation protocol was used, including 5 ns REST2 production simulations. The Δ*G* values were calculated for each leg using the multistate Bennett acceptance ratio (MBAR) method.^41^

### Molecular Docking

The ligands were prepared using the LigPrep tool of Schrödinger, Inc. at pH = 7 ± 1. Docking was performed with the Glide tool of Schrödinger Inc. with core constraints on the LIG5 coordinates from PDB: 8DEY crystal structure and AutoDock Vina 1.1.2.^42^ The protein structure and the ligands were preprocessed with AutoDockTools,^43^ nonpolar hydrogens were merged, and Gasteiger charges were assigned. A grid box of 28 Å × 28 Å × 28 Å was centered on LIG5 with a spacing of 1 Å. The maximum number of binding modes was set to 20, the exhaustiveness of the global search to 10.

### Interaction Energy Calculations

The interaction energy (IntE) between two groups of atoms was calculated as a sum of pairwise nonbonded electrostatic and van der Waals energy contributions using the all-atom additive CHARMM C36m force field with a distance dielectric constant of 2. The energy values reported are statistical averages calculated among the conformations retrieved from the production MD simulations.

### MM/GBSA

Molecular mechanics with generalized Born and surface-area solvation (MM/GBSA) analysis was performed on the MD conformational ensembles using the gmx_MMPBSA module.^44,45^ To approximate binding affinities, the protein–ligand complex conformations were used. Free energies were calculated as

16 $$G=E_{\mathrm{int}}+E_{elec}+E_{vdW+}G_{pol}+G_{np}-TS$$

where the first three energy terms correspond to the molecular-mechanics internal, electrostatic, and van der Waals energy contributions, *G*_pol_ and *G*_np_ are the polar and nonpolar solvation free energies, *T* is the temperature, and *S* the entropy.^46^ The internal, the electrostatic, and the van der Waals energy terms were calculated using the AMBER99SB force field.^47^ The explicit solvent molecules were removed before the postprocessing, and the GB-Neck model^48^ was used to estimate the polar component of the solvation free energy while the nonpolar solvation free energy was obtained by the equation

17 $$G_{np}=\gamma \cdot SASA$$

where SASA corresponds to the solvent-accessible surface, and γ = 0.0072 kcal·Å^–2^·mol^–1^ is an empirical constant. The binding free energy differences can be calculated as

18 $${\Delta G}_{bind}={\left\langle G\left(PL\right)-G\left(P\right)-G\left(L\right)\right\rangle }_{PL}$$

where PL denotes the protein–ligand complex, P the protein, and L the ligand, and all terms are calculated based on the protein–ligand complex MD simulation.

### Pomalidomide Scaffold Substructure Search

A substructure search of the pomalidomide core, as well as the core of five more compounds (Table 1) that are known to bind CRBN, was performed to find new commercially available potential glues of the CRBN/IKZF2 complex. For the substructure search, the Molecules as a Service (MaaS) web service of Orion, 2022 OpenEye Scientific Software, Inc. (MaaS 2.0.2. OpenEye, Cadence Molecular Sciences, Santa Fe, NM. http://www.eyesopen.com) was used to search through the Enamine and ZINC libraries. Also, a substructure search with SciFinder was conducted to identify commercially available compounds bearing the five substructures. A total of 1327 compounds was identified from the search, which was subsequently prepared and docked to the CRBN/IKZF2 complex with the LigPrep and Glide tools of Schrodinger, Inc. The same docking protocol that was used for the FEP+ calculations was also applied here.

**Table 1:**
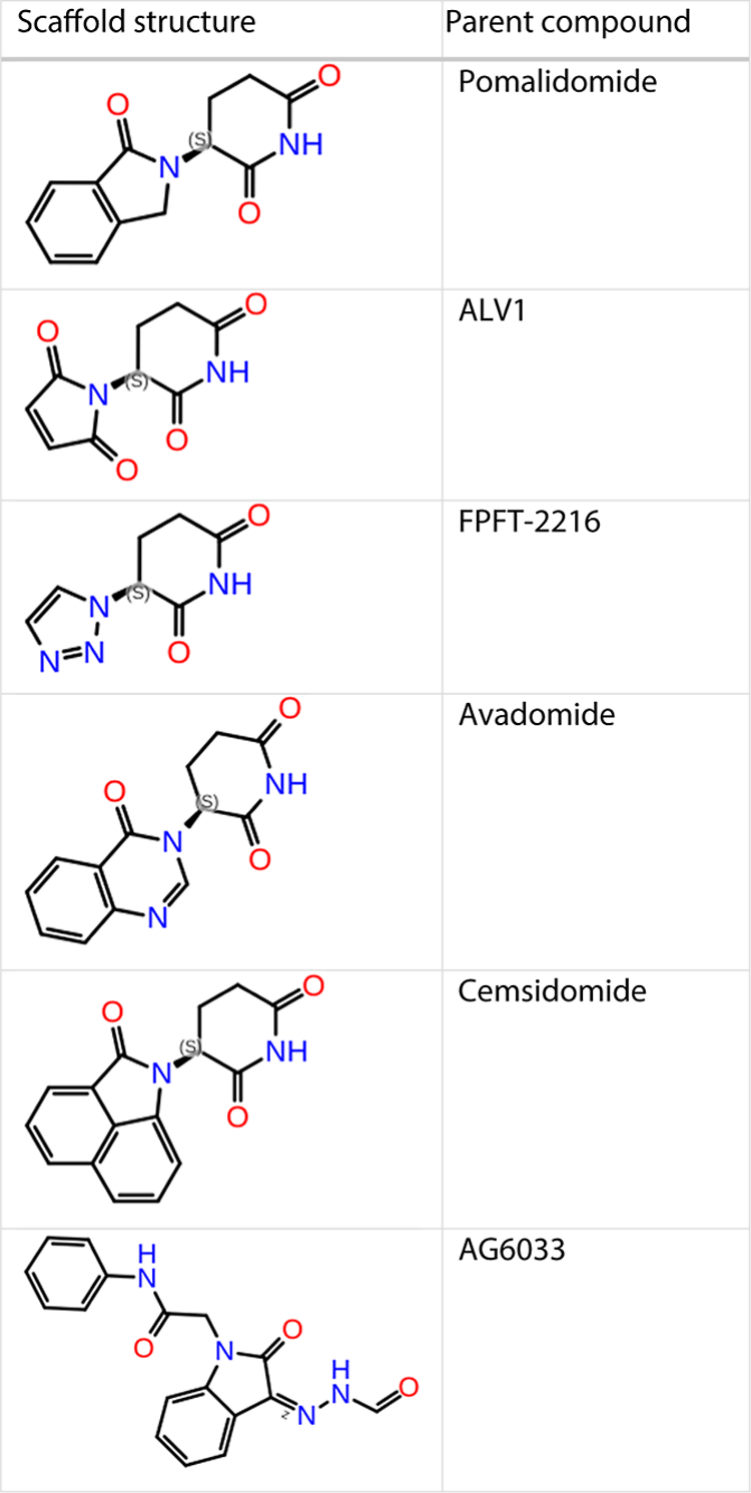
Scaffold Structures Used for the Substructure Search

## Supplementary Material



## Notes

Additional analysis of the complexes; additional interaction energy and cooperativity information; root-mean-square deviation analyses; list of best hit molecules with additional analyses (PDF)
