# Supplementary material for: Quantifying Cooperativity through Binding Free Energies in Molecular Glue Degraders
Source: J Chem Theory Comput. 2025 May 6;21(11):5712–23. doi: 10.1021/acs.jctc.5c00064 (PMC12159975; doi:10.1021/acs.jctc.5c00064)
Supplement: Supplementary file 1 [file ct5c00064_si_001.pdf]

# Supplementary Information

**Quantifying Cooperativity Through Binding Free Energies in Molecular Glue Degraders**

Balint Dudas[1,2], Christina Athanasiou[3], Juan Carlos Mobarec[3*], Edina Rosta[1*]

[1]Department of Physics and Astronomy, University College London, London WC1E 6BT, UK
[2]Laboratory of Computational Biology, National Heart, Lung, and Blood Institute, National Institutes of Health, Bethesda, MD 20892, USA
[3]Protein Structure and Biophysics, Discovery Sciences, R&D, AstraZeneca, Cambridge, UK
Correspondence: e.rosta@ucl.ac.uk, juancarlos.mobarec@astrazeneca.com

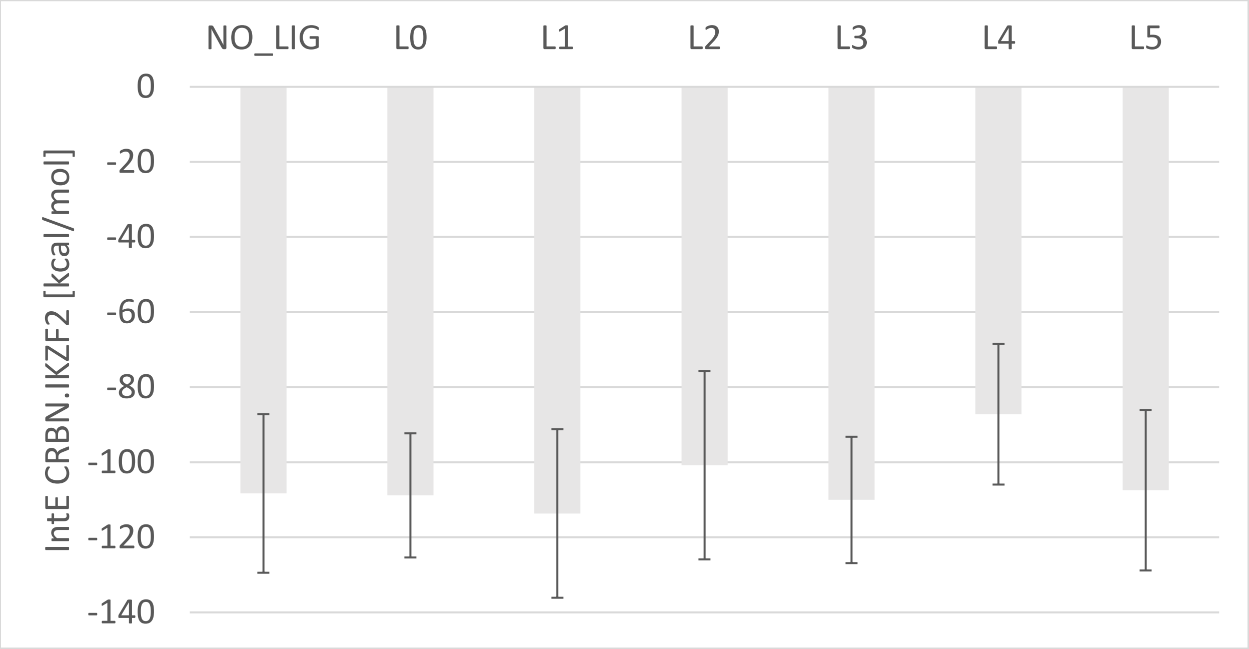


**Figure S1**: IntE between IKZF2 and CRBN in their dual complex and the ternary complexes with L0-L5 during the 3x100ns-long MD simulations.

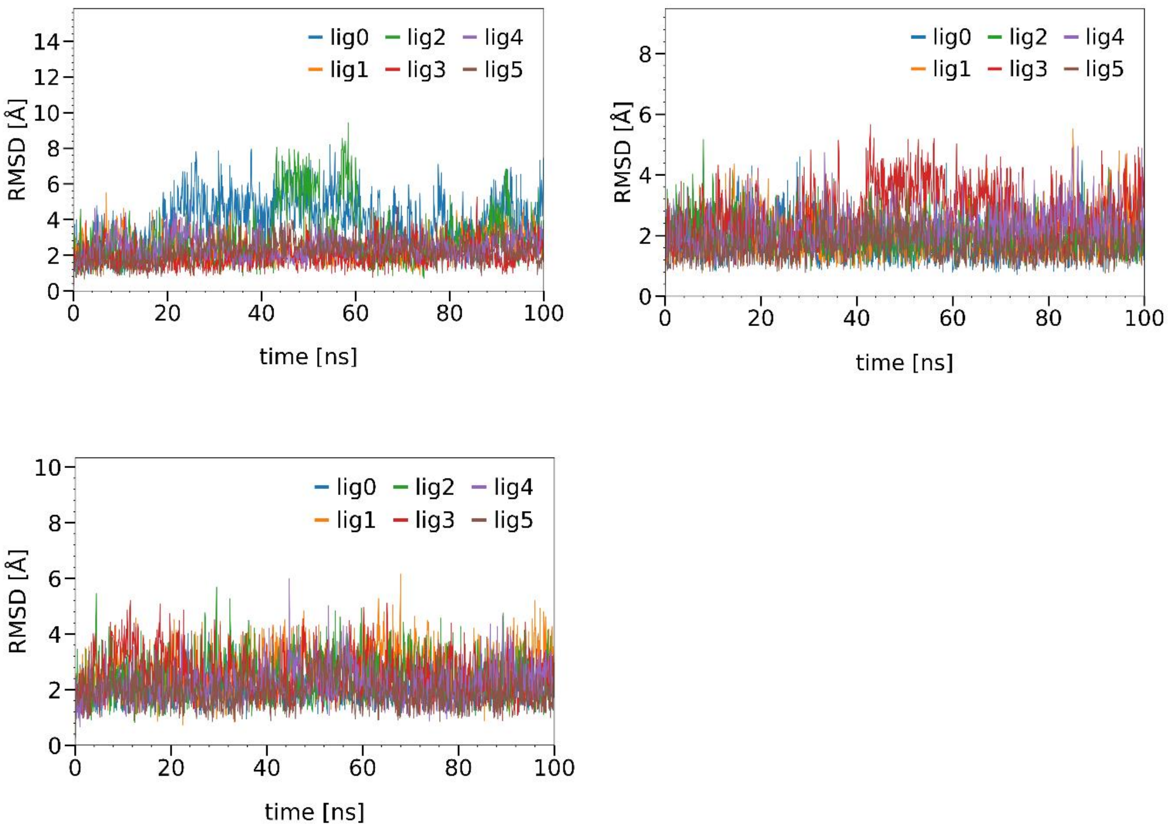


**Figure S2**: Backbone RMSD of the PPI formed between the IMiD-binding domain (IBD) of CRBN and ZF2 of IKZF2, over the 3x100ns-long MD simulations.

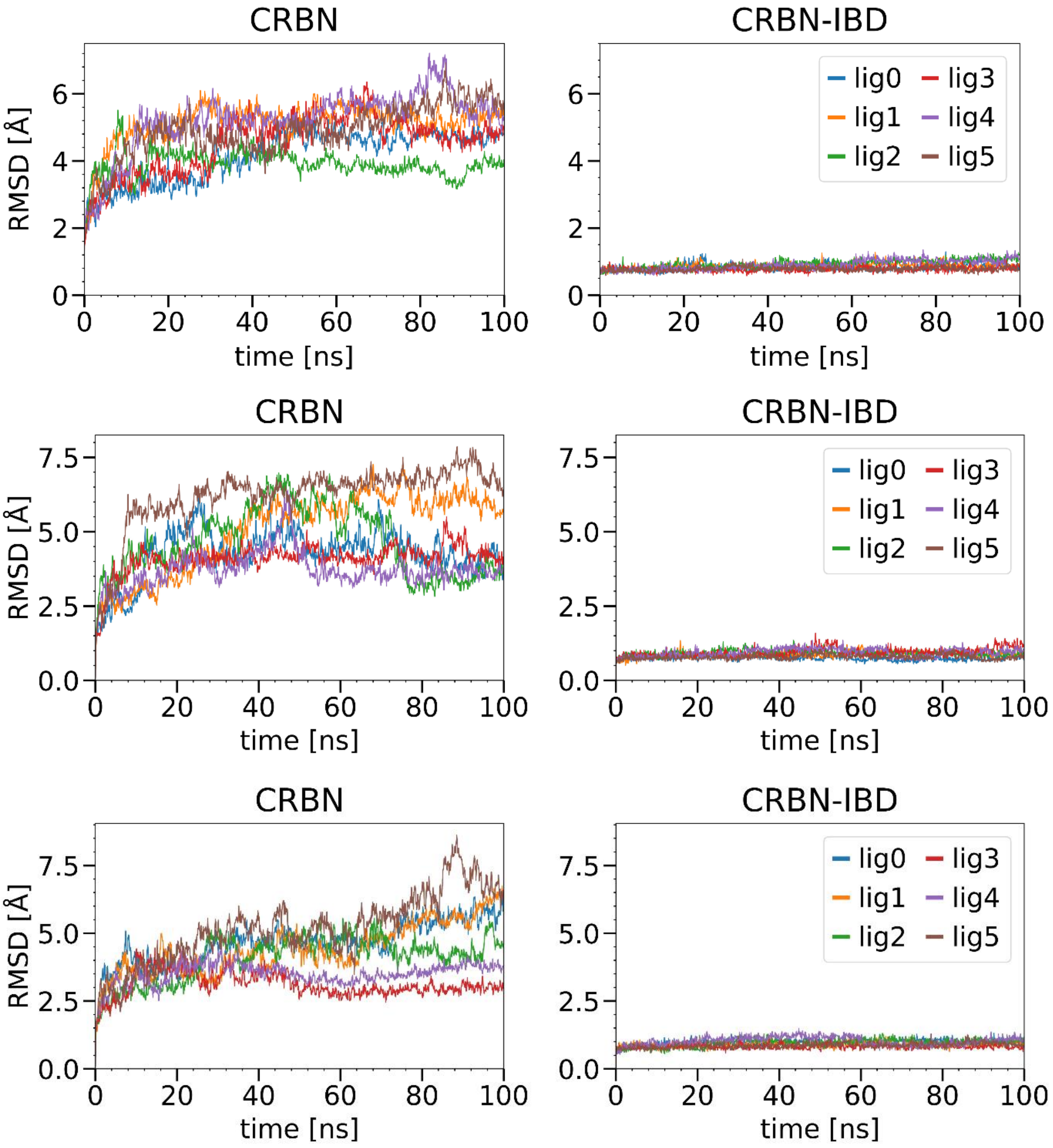


**Figure S3**: RMSD of the CRBN and its IMiD-binding domain (IBD) over the 3x100ns-long MD simulations.

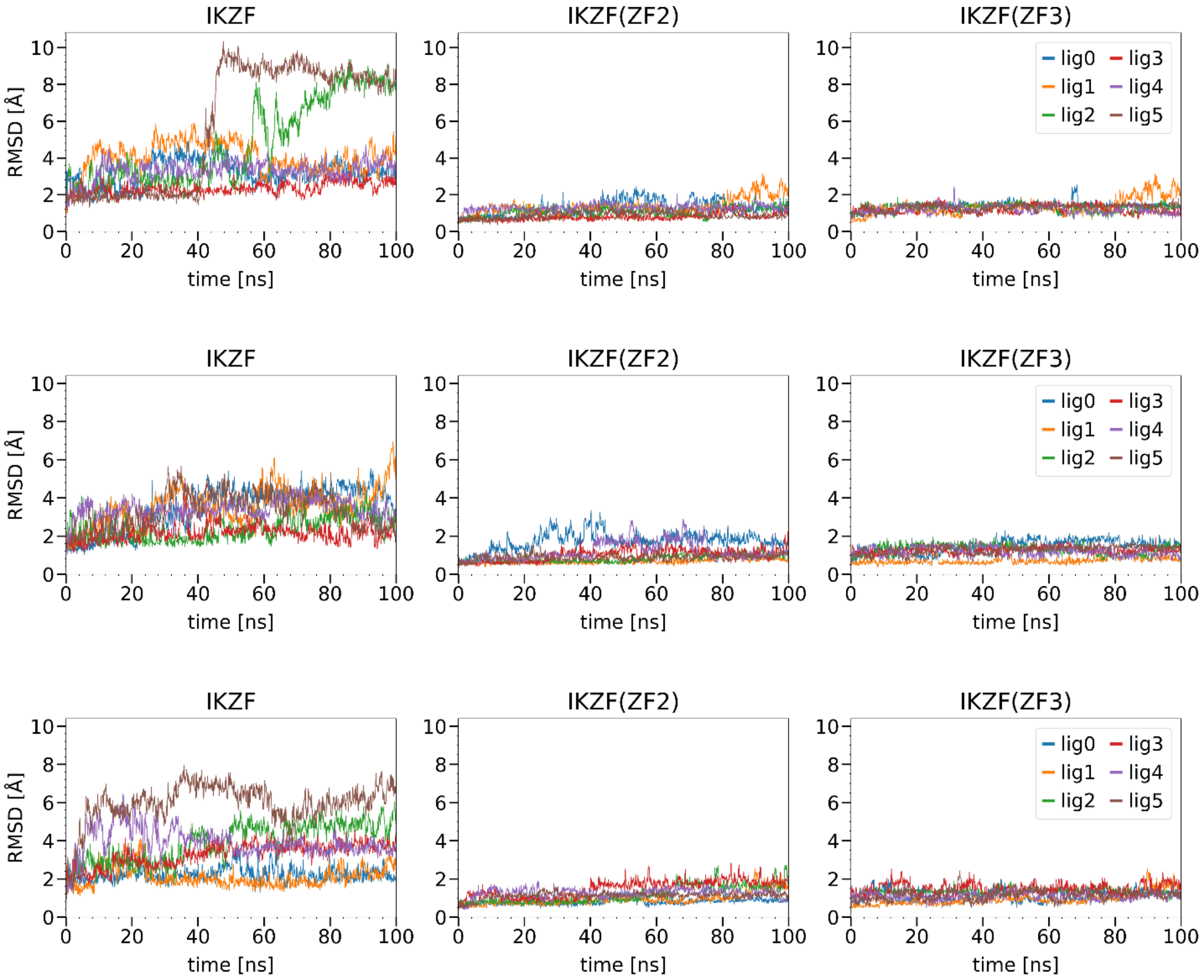


**Figure S4**: RMSD of the IKZF2 and its zinc finger 2 (ZF2) and zinc finger 3 (ZF3) over the 3x100ns-long MD simulations.

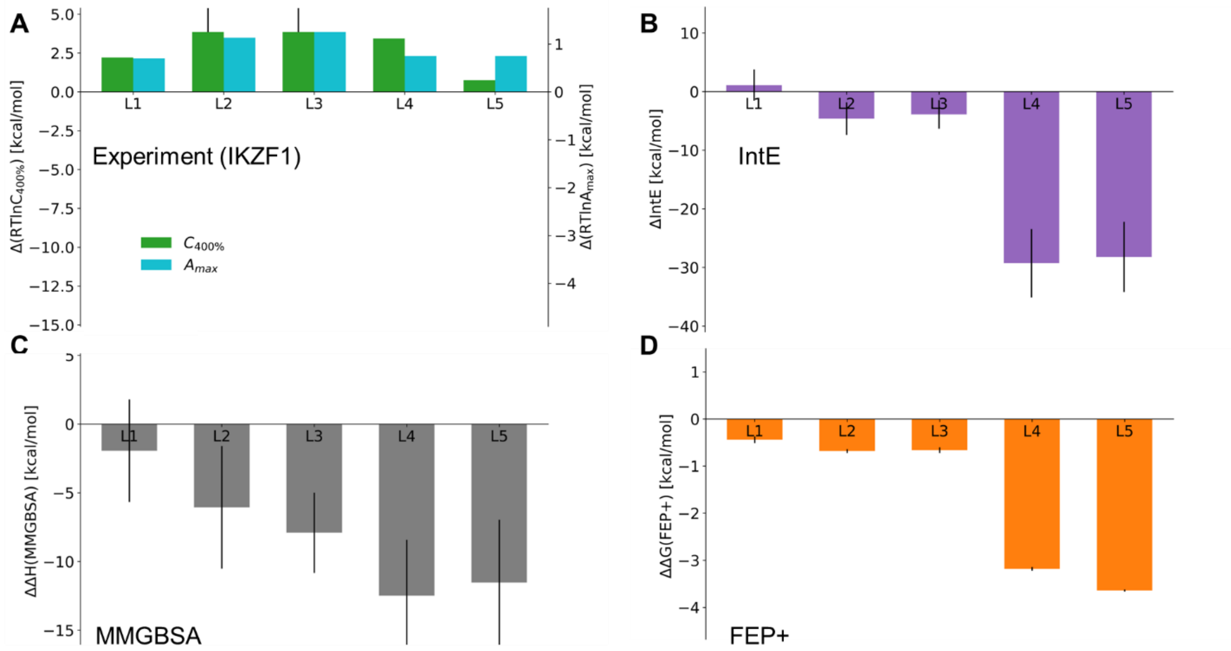


**Figure S5**: Relative binding affinity to CRBN.IKZF2(H141Q) of L1-L5 with respect to L0. A) Experimental $A_{max}$ values (cyan) and concentrations corresponding to 400% IKZF1 CRBN recruitment (green), transformed to free energy-like quantities (by taking RTlnC). B) Interaction energy and C) MM-GBSA enthalpy calculated from the 100ns-long MD simulations of the ternary complexes. D) Binding free energy calculated by FEP+ ligand perturbation simulations.

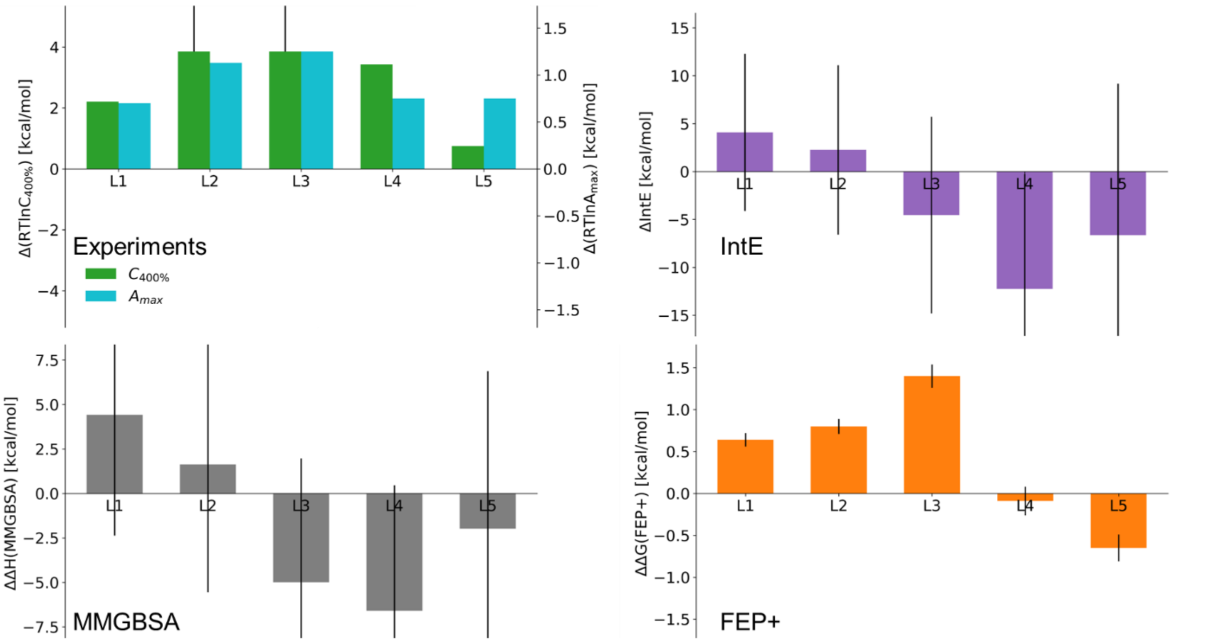


**Figure S6**: Relative cooperativity effects in the CRBN.IKZF2(H141Q).LIG complexes. For comparison, A) Experimental $A_{max}$ values (cyan) and concentrations corresponding to 400% IKZF1 CRBN recruitment (green), transformed to free energy-like quantities (by taking RTlnC). Cooperativity effects calculated B) by interaction energy, C) MM-GBSA, and D) FEP+.

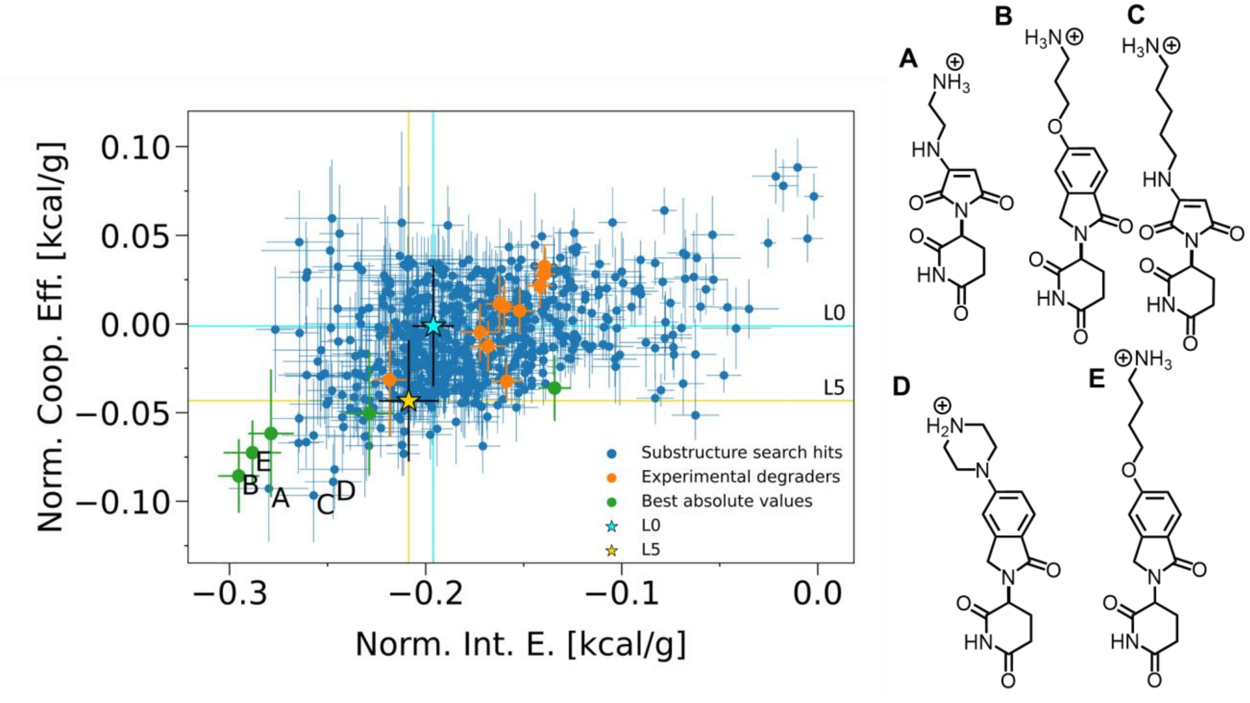


**Figure S7:** Predicted cooperativity and interaction energy for sub-structure search hits, experimental degraders and reference ligands L0 abd L5. The 2D structure of top ranked ligands are shown.

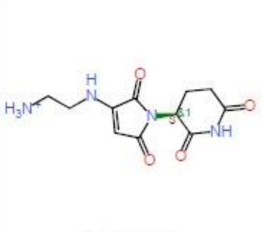
CSBRL0001

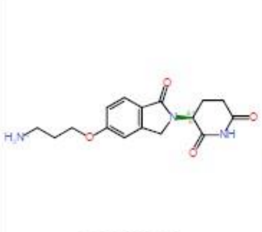
CSBRL0002

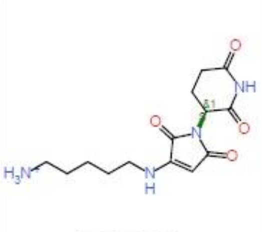
CSBRL0003

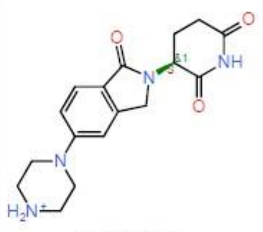
CSBRL0004

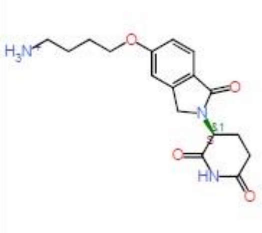
CSBRL0005

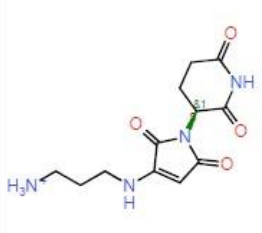
CSBRL06

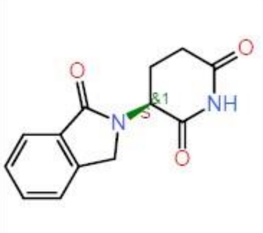
CSBRL07

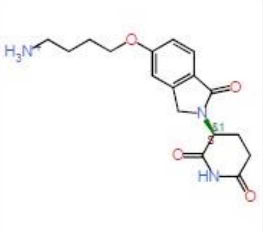
CSBRL0008

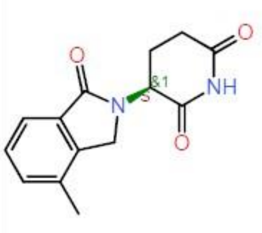
CSBRL09

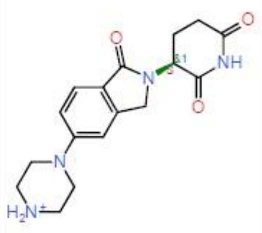
CSBRL0010

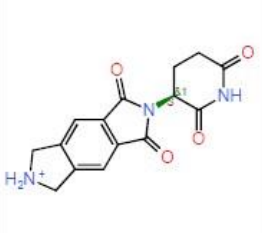
CSBRL0011

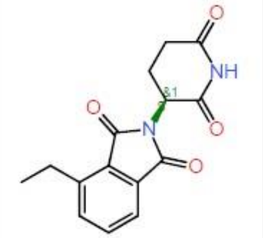
CSBRL0012

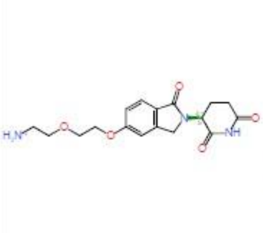
CSBRL0013

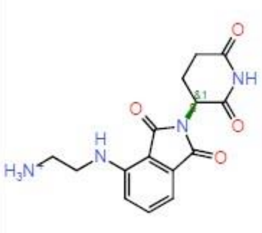
CSBRL0014

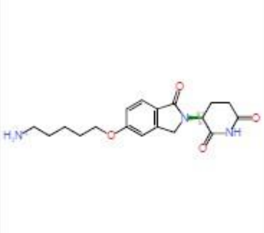
CSBRL0015

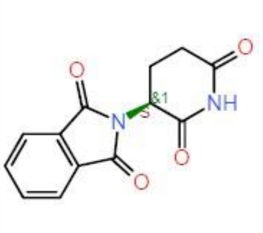
CSBRL0016

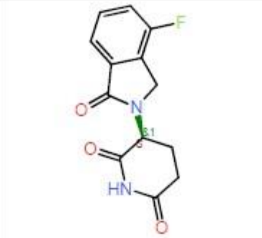
CSBRL0017

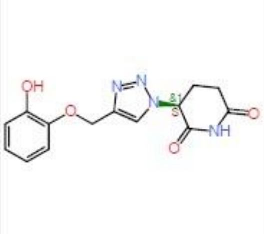
CSBRL0018

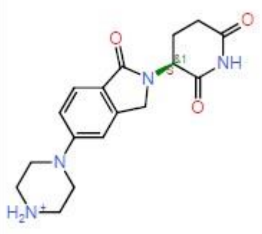
CSBRL0019

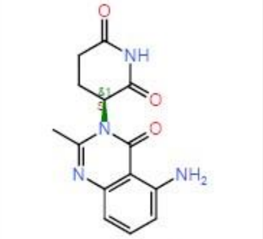
CSBRL0020

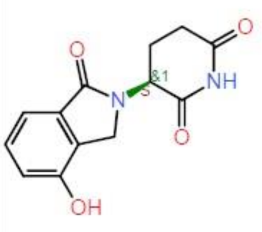
CSBRL0021

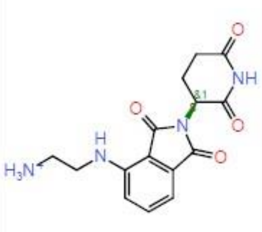
CSBRL0022

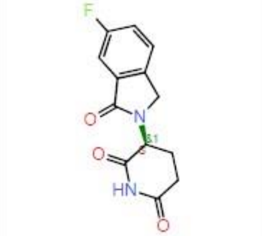
CSBRL0023

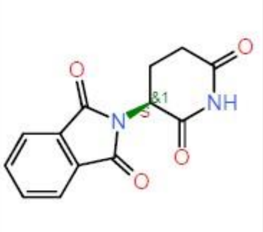
CSBRL0024

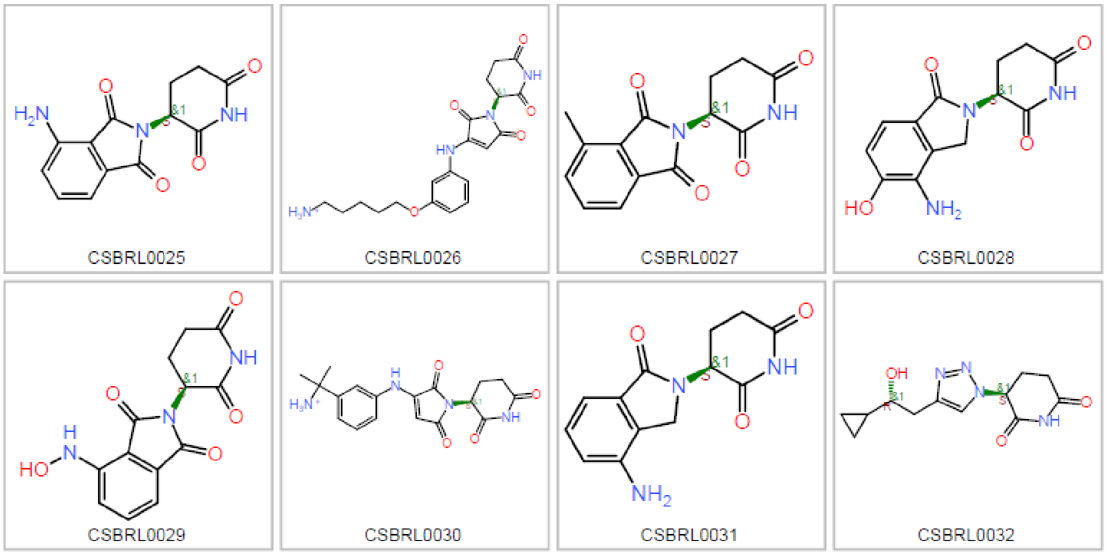


**Figure S8**: The 32 best hits (26 unique molecules) from the virtual screening.

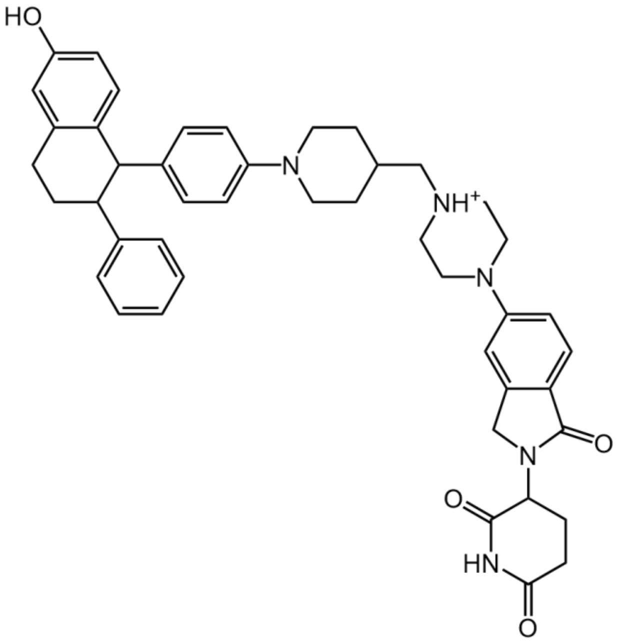

**Figure S9:** A top-ranked molecule from the unnormalized analysis that was omitted from further analysis due to its large size. This molecule is marked with a # in Fig. 7.

**Table S1**: Relative binding affinity and cooperative effect of the 26 best hits from the virtual screening campaign calculated by ligand FEP simulations using L0 (pomalidomide) as reference. The molecules are ranked based on their cooperative effect, L5 and L0 are also included in the list for comparison.

| Ligand | ΔΔG [kcal/mol] | ΔCE [kcal/mol] | Charge |
|---|---|---|---|
| L5 | -6.4±0.7 | -3.3±1.0 | 1 |
| CSBRL0004 | -4.3±0.6 | -2.2±1.0 | 1 |
| CSBRL0002 | -3.7±0.6 | -2.1±0.9 | 1 |
| CSBRL0033 | -4.0±0.5 | -2.0±0.9 | 1 |
| CSBRL0005 | -4.0±0.6 | -2.0±0.8 | 1 |
| CSBRL0034 | -3.6±0.4 | -2.0±1.0 | 1 |
| CSBRL0013 | -3.6±0.6 | -2.0±1.0 | 1 |
| CSBRL0015 | -3.8±0.6 | -1.8±0.9 | 1 |
| CSBRL0028 | -3.2±0.5 | -1.1±0.9 | 0 |
| CSBRL09 | -2.7±0.5 | -1.1±0.9 | 0 |
| CSBRL0017 | -1.7±0.5 | -1.1±0.9 | 0 |
| CSBRL0021 | -2.0±0.5 | -1.0±0.9 | 0 |
| CSBRL0030 | -1.3±0.8 | -0.8±1.1 | 1 |
| CSBRL0031 | -2.1±0.6 | -0.7±1.0 | 0 |
| CSBRL0003 | -0.6±0.8 | -0.5±1.1 | 1 |
| CSBRL07 | -1.4±0.4 | -0.5±0.9 | 0 |
| CSBRL0026 | -1.0±0.8 | -0.4±1.1 | 1 |
| CSBRL0023 | -1.0±0.4 | -0.4±0.8 | 0 |
| CSBRL0020 | 0.2±0.6 | -0.2±1.0 | 0 |
| CSBRL0029 | -0.2±0.4 | 0.0±0.9 | 0 |
| L0 | 0.0±0.4 | 0.0±0.8 | 0 |
| CSBRL06 | 0.3±0.7 | 0.1±1.0 | 1 |
| CSBRL0014 | 0.2±0.5 | 0.2±0.9 | 1 |
| CSBRL0012 | 0.0±0.5 | 0.4±0.9 | 0 |
| CSBRL0018 | 0.4±0.6 | 0.6±0.9 | 0 |
| CSBRL0024 | 1.4±0.6 | 0.7±0.9 | 0 |
| CSBRL0027 | 0.8±0.7 | 1.4±1.2 | 0 |
| CSBRL0001 | 3.7±0.8 | 1.5±1.1 | 1 |
| CSBRL0011 | 4.7±0.7 | 3.8±1.0 | 1 |
| CSBRL0032 | 5.3±0.6 | 5.3±1.2 | 0 |

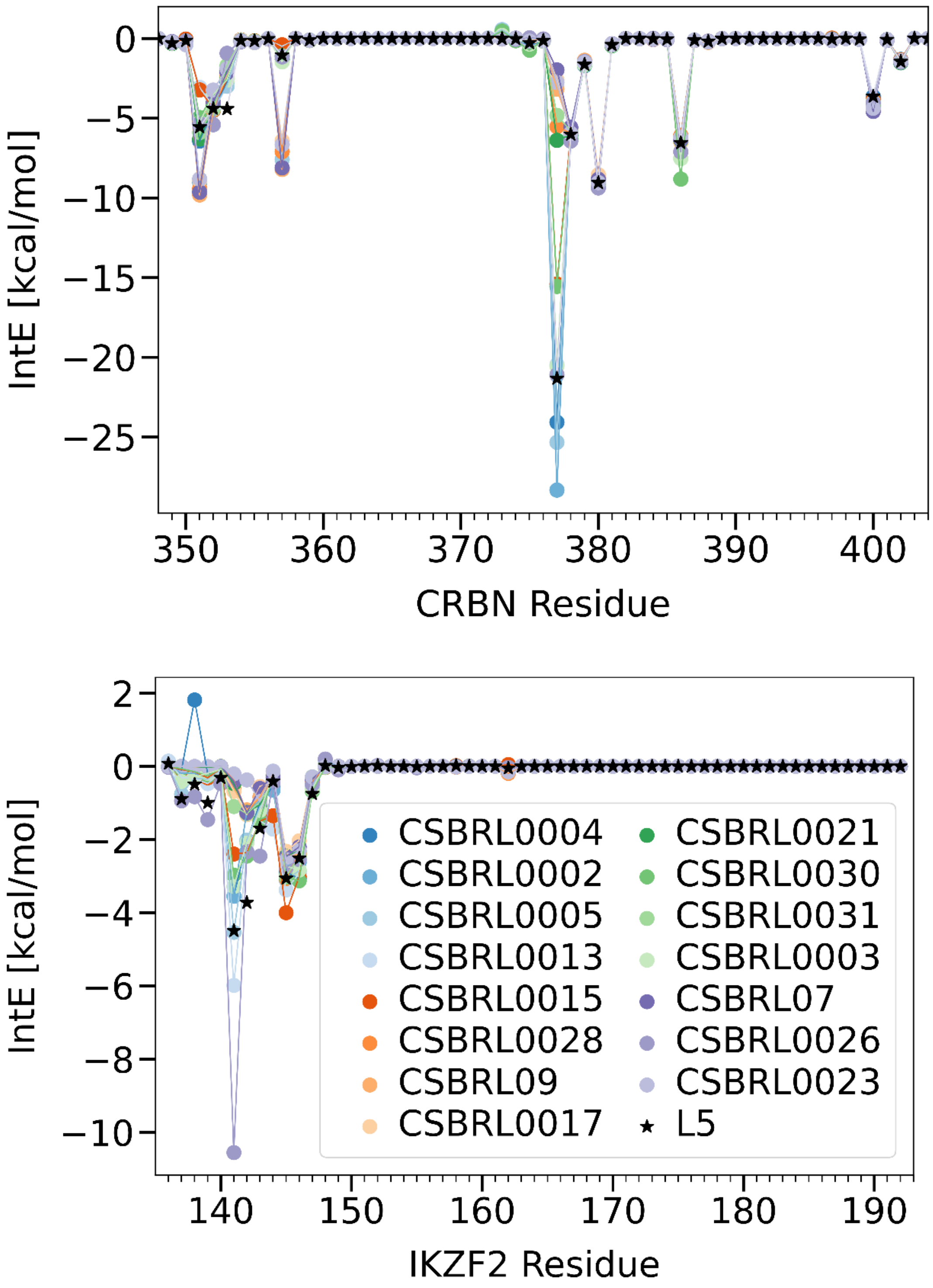


**Figure S10**: Interactions of the 15 best ligands predicted by FEP with CRBN (top) and IKZF2 (bottom) residues in the ternary complexes. The values shown are averages over the conformations originating from the MD simulations. The IntE values for L5 are also included as reference, shown in black stars.

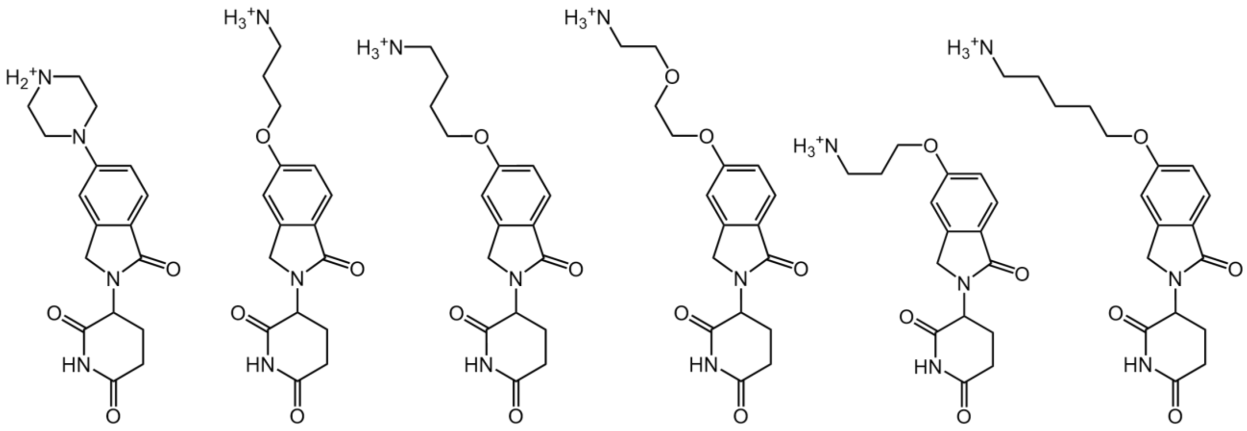

i ii iii,iv v vi vii

**Figure S11:** Top ranked molecules based on the FEP simulations. The following molecules are shown from Table S1: CSBRL0004 (i),CSBRL0002 (ii), CSBRL0033 (iii), CSBRL0005 (iv), CSBRL0034 (v), CSBRL0013 (vi), and CSBRL0015 (vii). Molecules i, iii, iv, and vi match molecules G, B, C, and A in Fig. 8, respectively.
